# Effects of Genetic and Physiological Divergence on the Evolution of a Sulfate-Reducing Bacterium under Conditions of Elevated Temperature

**DOI:** 10.1128/mBio.00569-20

**Published:** 2020-08-18

**Authors:** Megan L. Kempher, Xuanyu Tao, Rong Song, Bo Wu, David A. Stahl, Judy D. Wall, Adam P. Arkin, Aifen Zhou, Jizhong Zhou

**Affiliations:** a Institute for Environmental Genomics, University of Oklahoma, Norman, Oklahoma, USA; b Department of Microbiology and Plant Biology, University of Oklahoma, Norman, Oklahoma, USA; c School of Civil Engineering and Environmental Sciences, University of Oklahoma, Norman, Oklahoma, USA; d Department of Civil and Environmental Engineering, University of Washington, Seattle, Washington, USA; e Department of Biochemistry, University of Missouri, Columbia, Missouri, USA; f Environmental Genomics and Systems Biology Division, Lawrence Berkeley National Laboratory, Berkeley, California, USA; g Department of Bioengineering, University of California, Berkeley, California, USA; h Earth Sciences Division, Lawrence Berkeley National Laboratory, Berkeley, California, USA; i State Key Joint Laboratory of Environment Simulation and Pollution Control, School of Environment, Tsinghua University, Beijing, China; University of Montana; University of Pittsburgh

**Keywords:** *Desulfovibrio vulgaris*, evolutionary biology, stress adaptation, temperature stress

## Abstract

Adaptation via natural selection is an important driver of evolution, and repeatable adaptations of replicate populations, under conditions of a constant environment, have been extensively reported. However, isolated groups of populations in nature tend to harbor both genetic and physiological divergence due to multiple selective pressures that they have encountered. How this divergence affects adaptation of these populations to a new common environment remains unclear. To determine the impact of prior genetic and physiological divergence in shaping adaptive evolution to accommodate a new common environment, an experimental evolution study with the sulfate-reducing bacterium Desulfovibrio vulgaris Hildenborough (DvH) was conducted. Two groups of replicate populations with genetic and physiological divergence, derived from a previous evolution study, were propagated in an elevated-temperature environment for 1,000 generations. Ancestor populations without prior experimental evolution were also propagated in the same environment as a control. After 1,000 generations, all the populations had increased growth rates and all but one had greater fitness in the new environment than the ancestor population. Moreover, improvements in growth rate were moderately affected by the divergence in the starting populations, while changes in fitness were not significantly affected. The mutations acquired at the gene level in each group of populations were quite different, indicating that the observed phenotypic changes were achieved by evolutionary responses that differed between the groups. Overall, our work demonstrated that the initial differences in fitness between the starting populations were eliminated by adaptation and that phenotypic convergence was achieved by acquisition of mutations in different genes.

## Abstract (precis)

Improving our understanding of how previous adaptation influences evolution has been a long-standing goal in evolutionary biology. Natural selection tends to drive populations to find similar adaptive solutions for the same selective conditions. However, variations in historical environments can lead to both physiological and genetic divergence that can make evolution unpredictable. Here, we assessed the influence of divergence on the evolution of a model sulfate-reducing bacterium, Desulfovibrio vulgaris Hildenborough, in response to elevated temperature and found a significant effect at the genetic but not the phenotypic level. Understanding how these influences drive evolution will allow us to better predict how bacteria will adapt to various ecological constraints.

## INTRODUCTION

Since the time of Darwin, evolutionary biologists have attempted to improve our understanding of the underlying processes that shape evolutionary outcomes. Numerous studies have documented the importance of adaptation via natural selection and its tendency to push evolution to repeatable outcomes, especially when populations experience identical conditions (1, 2). However, when populations experience novel environments, genetic and phenotypic differences typically arise that can lead to divergent evolution (2–5). In nature, populations are likely to face a variety of environmental conditions and the extent to which previous divergence will be reflected at the genomic level and affect subsequent evolution remains unclear. Beneficial mutations accumulated in geographically isolated populations may constrain or promote adaptation when conditions change (6, 7). For example, a mutation conferring an adaptive phenotype in one genetic background may not confer a similar phenotype in an organism derived from the same ancestor but with a different genetic background, due to epistasis or complex interactions among different genes and/or mutations (8). Therefore, it is difficult to predict whether the history of a population and the previously acquired genetic differences will promote further divergence or if adaptation will lead to convergent changes when the populations experience the same conditions.

The effect of historical contingency on evolutionary outcomes has been intensely debated for years (reviewed in reference 2). Stephen J. Gould proposed the idea of “replaying life’s tape,” stating that if one were to go back in time and let evolution repeat itself, we would see a completely different outcome (9). As this type of evolutionary experiment is obviously impossible under natural settings, other approaches have been developed to experimentally address this topic. Microbial evolution studies provide several benefits that allow researchers to address issues which are not resolvable by studying organisms in a natural setting (3, 10). With microorganisms, the environment can be controlled and manipulated, thousands of generations can be acquired in a short amount of time, and samples can be cryopreserved and later revived for comparative investigations between populations from different generations (10). A simple approach to evaluate the influence of history and prior divergence on evolution in the laboratory is to use a two-phase experimental design. These studies typically evolve groups of replicate populations, derived from a single ancestor, under two or more sets of conditions for hundreds to thousands of generations before evolving all the populations under a novel set of conditions. The majority of previous studies designed in this manner evaluated the effect of historical contingency during evolution of bacteria (5, 11–14); simple eukaryotes, including yeast and a dinoflagellate (15–18); and viruses (19, 20). These studies revealed the tension between convergence and divergence and how the data derived from both depend on which traits are measured and the strength of selection. For instance, when populations that had previously diverged both phenotypically and genetically were evolved under the same new environment, signs of convergence were observed if traits strongly associated with fitness were measured (2, 5). However, whether these convergent traits were reflected at the genomic level remains unclear.

Wright’s metaphor of the adaptive landscape is commonly used to visualize how populations converge or diverge during evolution (21, 22). The adaptive landscape can be represented as a topographical map of genotype space where peaks and valleys represent areas of high and low fitness, respectively. Adaptation leads a population to climb to a peak with a high fitness value. The landscape can be smooth, with only one available peak, or rugged, with multiple peaks of various heights. Epistasis can cause the nature of a fitness effect of a mutation to be contingent upon the presence of other mutations, which would result in a landscape of various peaks separated by valleys of low fitness (23). Populations that initially differed genetically might not be able to reach the same fitness peak if the process includes traversing a valley with low fitness. Therefore, rugged landscapes tend to support the divergence of populations as they climb to different fitness peaks (2, 22).

Most experimental evolution studies have focused on model organisms such as Escherichia coli and yeast (Saccharomyces cerevisiae), and yet the intricacies of genetic adaptation are likely quite different for distantly related microorganisms due to the specific metabolic and regulatory pathways present. Desulfovibrio vulgaris Hildenborough (DvH) is an obligate anaerobe that has been extensively studied as a model sulfate-reducing bacterium (SRB) and is known to play an important role in global sulfur and carbon cycles (24). SRB are environmentally and industrially significant as the production of sulfide can cause souring of oil and corrosion in the petroleum industry (25). However, SRB can also be utilized to reduce toxic heavy metals in hazardous waste-contaminated sites (26–28). Here, we investigated whether DvH populations that had diverged genetically and physiologically would continue to diverge or if signatures of genetic convergence would emerge when evolved under identical conditions. This work employed two groups of populations that had diverged during a previous evolution study while adapting to either salt (NaCl) stress or nonstress conditions (29, 30). These populations were then evolved in a second, novel environment (elevated temperature). Temperature is a ubiquitous ecological factor that influences where a species can live and affects a myriad of cellular processes. A third group of populations that was founded from the parent strain that was used for both the salt-stressed and nonstressed evolved-population experiments described above was evolved in elevated temperature as well. Thus, three groups of six populations that each had a clonal founder were evolved for 1,000 generations at 41°C.

We hypothesized that all three groups of populations would achieve improved fitness under conditions of elevated temperature but that the underlying genetic changes would be influenced by the historical environment. We expected that the majority of mutations acquired under conditions of elevated temperature would be single nucleotide changes as shown in previous evolution studies in both DvH and E. coli (30–32). Additionally, increased expression in genes involved in protein turnover and chaperone activity and decreased expression of genes involved in energy production and conversion, nucleotide transport and metabolism, translation, ribosomal structure, and biogenesis have been observed in the response of DvH to an elevated growth temperature (33). Evolution studies of E. coli performed under conditions of elevated temperature identified mutations in genes affecting RNA polymerase activity, chaperonin expression, and cell wall synthesis (31, 32, 34).Therefore, we expected that genes in these functional groups would acquire mutations during evolution under conditions of elevated temperature and yet that different mutations would be selected at the gene level in a manner depending on the prior genetic divergence.

## RESULTS

### Overview of experimental design.

**FIG 1:**
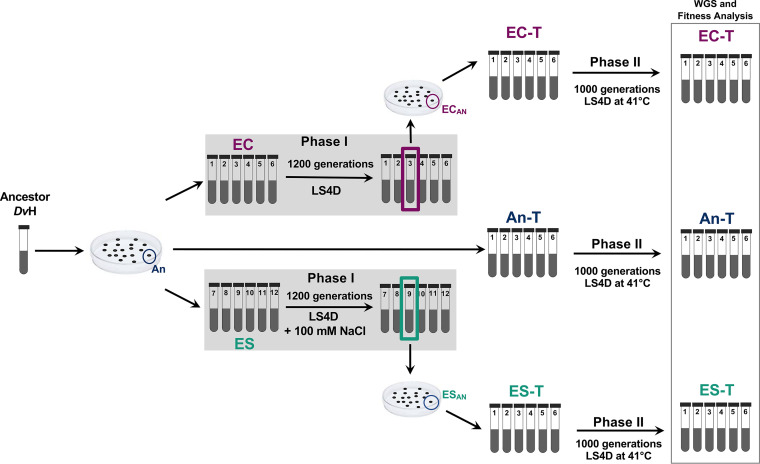
Overview of experimental design. Six ancestor populations with no prior experimental evolution (An-T 1 to An-T 6), six evolved control populations with 1,200 generations maintained under control conditions (EC-T 1 to EC-T 6), and six evolved salt populations with 1,200 generations maintained under elevated-salt conditions (ES-T 1 to ES-T 6) were propagated for 1,000 generations at an elevated temperature (41°C). In addition to the An, EC_AN_, and ES_AN_ clonal isolates, the genomes of all 18 populations after phase II were resequenced. WGS, whole-genome sequencing.

In a previous study, 12 nearly isogenic populations were initialized from a single ancestral clonal isolate of DvH and propagated for 1,200 generations under either control conditions or elevated-salt conditions (29). After the completion of this initial evolution (referred to as phase I), clones were picked from each group and tested for their ability to survive salt stress. The best-performing clone from each group (group EC [evolved control] 3–10 and group ES [evolved salt] 9–11, referred to here as EC_AN_ and ES_AN,_ respectively, for clarity) was further characterized, and the divergence in growth rates and salt tolerances of EC_AN_ and ES_AN_ was investigated (29, 30). EC_AN_ and ES_AN_ were used as founding clones of phase II of experimental evolution. Six populations initiated from EC_AN_ were named EC-T (EC-temperature) 1–6, six populations initiated from ES_AN_ were named ES-T (ES-temperature) 1–6, and six populations from the original ancestor were named An-T 1 to An-T 6 (where “An” represents “ancestor” and “T” represents temperature). All 18 populations were propagated for 1,000 generations at 41°C (phase II; Fig. 1). Thus, three groups, each containing six genetically identical replicates, were subsequently subjected to evolution under the same novel set of conditions in phase II.

### Changes in growth rate and fitness after phase II evolution.

**FIG 2:**
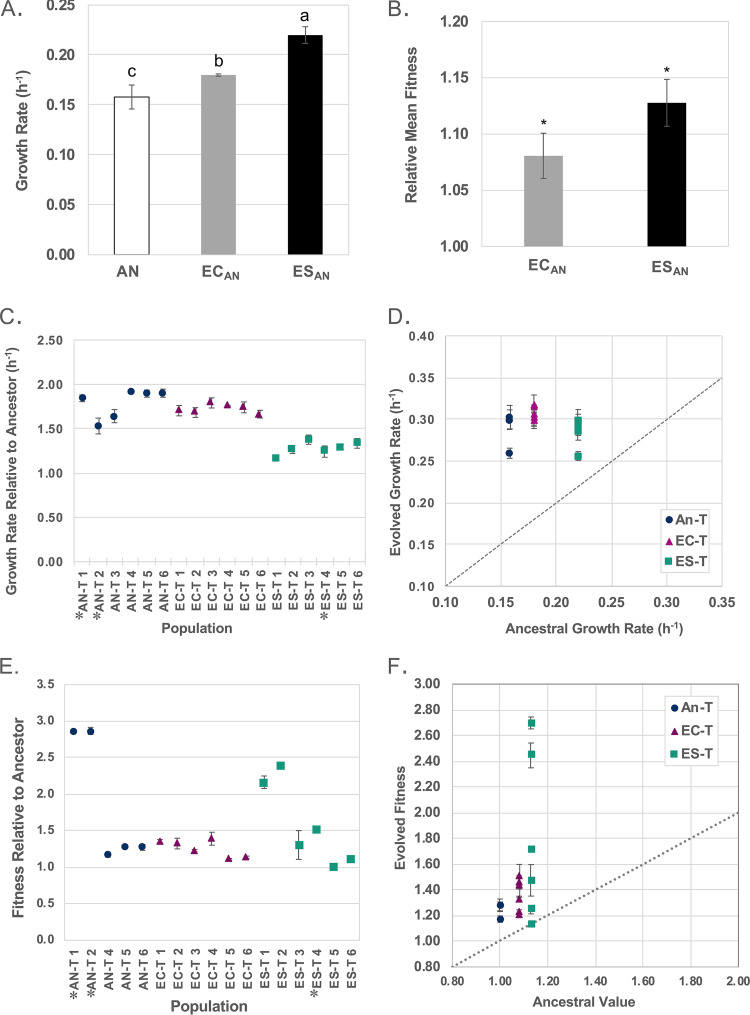
(A) Growth rates of An, EC_AN_, and ES_AN_ at 41°C. Bars with different letters differ significantly (*P* *≤* 0.05, one-way ANOVA, Tukey’s pairwise comparison). (B) Relative fitness of EC_AN_ and ES_AN_ calculated from head-to-head competition with An. *, *P* ≤ 0.05 (one-tailed *t* test where the null hypothesis is that mean fitness equals 1.0). (C) Growth rate of populations after 1,000 generations at 41°C relative to the corresponding ancestor. Asterisk (*) indicates a mutator population. (D) Evolved mean growth rate versus the corresponding ancestral mean growth rate of the nonmutator populations. (E) Fitness of populations after 1,000 generations at 41°C relative to the corresponding ancestor. An asterisk (*) indicates a mutator population. (F) Evolved mean fitness versus the corresponding ancestral mean fitness of the nonmutator populations. An-T 3 was excluded from fitness analysis (E and F) due to the lack of a fixed marker mutation. Data in panels A to F represent means of results from three replicates, and all error bars represent standard deviations (SD).

To evaluate the phenotypic changes seen after evolution at the elevated temperature, both maximum growth rate and fitness were measured for the three groups of evolved populations and their relevant ancestors. Here, we define fitness as the ability of one population to survive and reproduce in the given environment when competing for resources against a second population. Both EC_AN_ and ES_AN_ had higher growth rates and fitness in the novel environment (41°C) than the original ancestor (An, the parent strain corresponding to the prior evolutionary treatment) at the beginning of phase II (Fig. 2A and B). These results demonstrated that the previous adaptations of both EC_AN_ and ES_AN_ conferred competitive advantages that were evident at the beginning of phase II.

After phase II, the maximum growth rates of all evolved populations increased, indicating adaptation to the novel 41°C environment (Fig. 2C and D). The grand mean growth rate of the populations had significantly increased (*t* test, *P* < 0.001 [with and without mutators]) compared to that of the corresponding ancestors, indicating the significant influence of adaptation (Table 1). Additionally, the changes in growth rates were dependent on the prior environments in which ES_AN_ and EC_AN_ were propagated in phase I, as the effect of previous genetic and phenotypic divergence in EC-T and ES-T was significant in testing performed with nested analysis of variance (ANOVA) (F_10,24_ = 6.21, *P* = 0.03 with all populations; F_9,22_ = 4.9, *P* = 0.05 without mutators). In contrast, changes in growth rate were not influenced by within-group differences, indicating the effect from chance was not significant (L ratio = 0.05, *P* = 0.81 with all populations; L ratio = 0.55, *P* = 0.46 without mutators). These results demonstrated that the prior evolutionary condition influenced the changes in growth rate during adaptation to elevated temperature.

**TABLE 1:** Mean growth rate of each group after 1,000 generations at 41°C

Group	Grand mean (± SE)	*P*^*a*^
An-T	0.282 (0.004)	<0.001
An-T (no mutators)	0.290 (0.005)	<0.001
EC-T	0.307 (0.003)	<0.001
ES-T	0.288 (0.004)	<0.001
ES-T (no mutators)	0.290 (0.006)	<0.001

a One-tailed *t* test. The null hypothesis is that the mean growth rate equals the ancestral growth rate for each group (An = 0.158, EC_AN_ = 0.180, and ES_AN_ = 0.220).

Fitness was measured by allowing the evolved populations to directly compete head to head with the original ancestor under phase II conditions (41°C). Competitors were mixed in a 1:1 ratio based on measurements of optical density at 600 nm (OD_600_). Sequencing results showed that the measured starting percentages of the evolved population at time 0 h ranged from 42% to 57% of the total inoculum. An-T 3 was excluded from fitness analyses due to the lack of a nonpolymorphic derived fixed mutation to distinguish it from the ancestor. All but one population (ES-T 5) had increased fitness compared to the corresponding ancestor (Fig. 2E and F). Interestingly, ES-T 5 showed an increased growth rate like the other populations in the ES-T group but no increase in fitness, suggesting that factors other than growth rate contributed to overall fitness (Fig. 2E and F). The contributions of both adaptation (*t* test, *P* = 0.001 for the An-T group and *P* < 0.001 for the EC-T and ES-T groups with all populations; *P* < 0.001 for the An-T and EC-T groups and *P* = 0.002 for the ES-T group without mutators) (Table 2) and chance (nested ANOVA L ratio = 69.35 and *P* < 0.001 with all populations; L ratio = 63.76 and *P* < 0.001 without mutators) to the improved fitness were significant. However, there was no significant difference between the EC-T and ES-T groups (nested ANOVA F_10,24_ = 2.51 and *P* = 0.14 with all populations; F_9,22_ = 2.21 and *P* = 0.171 without mutators), suggesting that the competitive advantages that EC_AN_ and ES_AN_ gained in phase I did not significantly contribute to the improved fitness of the populations. Fitness was quite variable within the ES-T group due to the fact that two populations, ES-T 1 and ES-T 2, had much higher fitness than any of the other populations in the ES group. Overall, our results indicated that the prior states of genetic and physiological divergence did not significantly affect the fitness improvements observed.

**TABLE 2:** Mean fitness of each group after 1,000 generations at 41°C

Group	Grand mean (± SE)	*P*^*a*^
An-T^*b*^	1.89 (0.213)	0.001
An-T^*b*^ (no mutators)	1.24 (0.019)	<0.001
EC-T	1.36 (0.029)	<0.001
ES-T	1.78 (0.145)	<0.001
ES-T (no mutators)	1.80 (0.174)	0.002

a One-tailed *t* test. The null hypothesis is that the fitness level equals 1.0.

b An-T 3 was not included in fitness analysis.

### Overall genetic similarity between populations.

**FIG 3:**
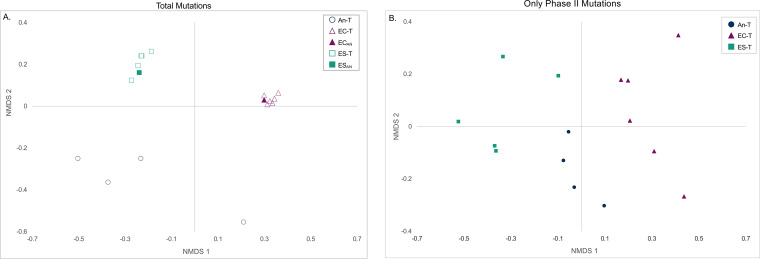
Nonmetric multidimensional scaling plot of population mutation profiles at the gene level (nonsynonymous mutations) based on the Bray-Curtis dissimilarity for (A) all mutations acquired for each population (stress = 0.07) and (B) only mutations acquired during phase II (stress = 0.15). Populations were significantly more genetically similar within groups than between groups for both total mutations and only phase II mutations (permutation analysis of variance, *P* = 0.001).

To evaluate the overall genetic similarity between the evolved populations, Bray-Curtis dissimilatory data were calculated at the gene level (35). The differences between the nonmutator populations from the different groups can be viewed in a nonmetric multidimensional scaling (NMDS) plot (Fig. 3). Permutation analysis of variance indicated that the populations were more genetically similar within groups than between groups if the total mutational profiles of the populations were considered (*P* = 0.001; Fig. 3A) or if only the phase II mutations were considered (*P* = 0.001; Fig. 3B). There were significant differences in genetic similarity between all three groups seen in the total mutational profiles (permutation analysis of variance, An-T versus EC-T, *P* = 0.009; An-T versus ES-T, *P* = 0.012; EC-T versus ES-T, *P* = 0.004) or in just phase II mutations (permutation analysis of variance, An-T versus EC-T, *P* = 0.003; An-T versus ES-T, *P* = 0.021; EC-T versus ES-T, *P* = 0.004). These results implied overall genetic divergence among the three groups of populations after phase II evolution. Next, we analyzed the individual mutations to determine the effect of genetic divergence derived from phase I evolution on the genetic changes in the phase II environment. Here, we use the term “convergence” to indicate similar genetic changes in populations that started from genetically different ancestors and the term “parallelism” to refer to the accumulation of similar genetic changes in populations that started from the same ancestral genotype (2).

### Mutations acquired during phase I of evolution.

The ancestor (An), EC_AN_, and ES_AN_ were previously sequenced and described in detail (30). Our DvH ancestor (An) contained 22 mutations compared to the NCBI reference strain (see Table S1 in the supplemental material). Additionally, 11 polymorphic loci were identified in An (Table S2). After 1,200 generations in phase I, these loci were no longer polymorphic (Table S2). Six of these loci were mutated in EC_AN_, while the other five loci were mutated in ES_AN_. Therefore, at the beginning of phase II evolution, the starting populations in the EC-T group contained 14 single nucleotide variants (SNVs) and 1 indel and the populations in the ES-T group contained 9 SNVs and 2 indels, including the polymorphic derived mutations (Table S3).

10.1128/mBio.00569-20.2 TABLE S1 Preexisting mutations in the ancestor DvH strain compared to the reference Desulfovibrio vulgaris Hildenborough genome from NCBI (NC_002937). (The table was constructed based on data from A. Zhou, K. L. Hillesland, Z. He, W. Schackwitz, et al., ISME J 9:2360–2372, 2015, https://doi.org/10.1038/ismej.2015.45). Download Table S1, DOCX file, 0.01 MB. Copyright © 2020 Kempher et al. 2020 Kempher et al. This content is distributed under the terms of the Creative Commons Attribution 4.0 International license.

10.1128/mBio.00569-20.3 TABLE S2 Polymorphic loci in ancestral DvH and selected nucleotide in EC_AN_ and ES_AN_. (The table was constructed based on data from A. Zhou, K. L. Hillesland, Z. He, W. Schackwitz, et al., ISME J 9:2360–2372, 2015, https://doi.org/10.1038/ismej.2015.45). Download Table S2, DOCX file, 0.01 MB. Copyright © 2020 Kempher et al. 2020 Kempher et al. This content is distributed under the terms of the Creative Commons Attribution 4.0 International license.

10.1128/mBio.00569-20.4 TABLE S3 Mutations acquired by ES_AN_ and EC_AN_ during phase I of evolution. (The table was constructed based on data from A. Zhou, K. L. Hillesland, Z. He, W. Schackwitz, et al., ISME J 9:2360–2372, 2015, https://doi.org/10.1038/ismej.2015.45). Download Table S3, DOCX file, 0.01 MB. Copyright © 2020 Kempher et al. 2020 Kempher et al. This content is distributed under the terms of the Creative Commons Attribution 4.0 International license.

### Number of mutations and emergence of mutators during phase II of evolution.

**FIG 4:**
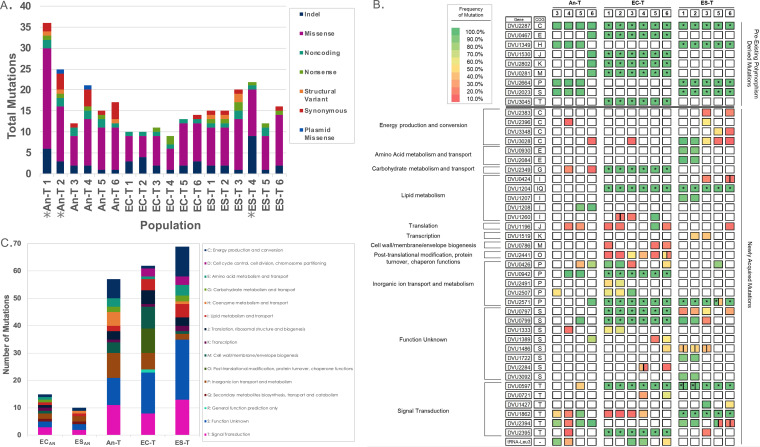
(A) Overview of the genetic changes in the 18 evolved populations of DvH after 1,000 generations at 41°C. An asterisk (*) designates a mutator population. (B) Summary of all genes (rows) that acquired mutations in two or more nonmutator populations (columns). Genes are grouped based on assigned COG groups. An asterisk (*) denotes those mutations that occurred during phase I of evolution in EC_AN_ and ES_AN_. Boxes are colored based on the frequency of the mutation (Table S4). Boxes divided down the middle represent two different mutations that occurred in the same gene. (C) Genetic changes based on COG designations for each gene that acquired a mutation in a nonmutator population for each group and the two ancestors EC_AN_ and ES_AN_.

At the end of phase II evolution, a total of 118 genes (1 of these genes was located on pDV, a 203-kb native plasmid) had acquired mutations that had risen to a frequency of ≥10% in at least one population (Table S4). Each population acquired 9 to 36 total mutations (Fig. 4A). The average numbers of mutations in the An-T, EC-T, and ES-T populations were 21, 11, and 17, respectively. Additionally, three populations (An-T 1, An-T 2, and ES-T 4) had the highest numbers of mutations and appeared to have developed a mutator phenotype. ES-T 4 has a mutation (variant frequency of 92.4%) in *mutL* (D. vulgaris 0483 [DVU0483]), which encodes a component of the mismatch repair system, and mutations in this gene have been previously shown to lead to a mutator phenotype in E. coli (36, 37). An-T 1 and An-T 2 contained mutations in DVU1515, corresponding to *dcm*, which encodes a putative type II DNA modification methyltransferase (C-5 cytosine specific; Table S4). An-T 1 had two missense mutations and one 12-bp insertion in *dcm*, and An-T 2 contained one missense mutation in *dcm* (Table S4). In E. coli, the methyltransferase encoded by *dcm* methylates the second cytosine of CCWGG sequences. Spontaneous deamination of the methylated cytosine converts it to thymine, resulting in C-to-T mutations that are normally repaired by the very-short-patch (VSP) repair system (reviewed in reference 38). Nine of the SNVs observed in An-T 1 were C-to-T mutations, while An-T 2 contained only two C-to-T mutations. The exact connection between the *dcm* mutations and an increased number of mutations in DvH populations remains to be determined. Taking the results together, we cannot confidently state that the mutations acquired in these populations were not affected by errors in DNA repair; therefore, they were excluded from our genetic analyses. With the removal of these populations, the total number of mutations acquired by genes during phase II of evolution in the 15 nonmutator populations was 91 (Table S4). The majority (53%, 48 of 91) of genes that had acquired a mutation during phase II were found in only one population. Of these, 14 genes were mutated in only one An-T population, 16 genes were mutated in only one ES-T population, and 18 genes were mutated in only one EC-T population (Table S4). The presence of these unique mutations in each population contributed to the overall genetic differences between the groups.

10.1128/mBio.00569-20.5 TABLE S4 Total mutations acquired for each population (variant frequency of ≥10%) during 1,000 generations at 41°C. Download Table S4, DOCX file, 0.08 MB. Copyright © 2020 Kempher et al. 2020 Kempher et al. This content is distributed under the terms of the Creative Commons Attribution 4.0 International license.

### Convergent mutations acquired during phase II evolution.

Mutations that occurred in two or more independently evolved populations and that reached a frequency of at least 10% were likely under positive selection given the large population size (∼10^7^ cells at transfer) (39–41). Here, mutations common in two or more groups of higher-temperature-evolved populations were classified as convergent mutations and those mutations that occurred in two or more populations within a single group were classified as parallel mutations. Convergent mutations could be classified in two categories. One category included mutations in ES_AN_ or EC_AN_ acquired in phase I and new mutations in these genes that occurred in phase II evolution. Since these genes were mutated during phase I under control or salt stress conditions and during phase II under conditions of elevated temperature, they likely represent mutations beneficial for adaptation to general lab growth conditions or nonspecific stress. Eight genes belonged to this category (Fig. 4B), with the majority of these genes mutated in EC_AN_ and then mutated in at least one population from another group (DVU0942, DVU2349, DVU0797, DVU0799, and DVU2395). One gene was mutated in ES_AN_ (DVU1862) and then mutated in at least one population of the EC-T group. Two additional genes were mutated in both ES_AN_ and EC_AN_ (DVU0597 and DVU2571) and then were mutated in at least one population of the An-T group. However, additional mutations were acquired in these two genes during phase II in the ES-T group. Additionally, of the 11 polymorphic loci present in An, 4 were mutated in An-T 3, An-T 4, and An T-5 and 5 were mutated in An-T 6 (Table S4).

The second category of convergent genetic changes included new mutations that occurred in phase II in two or more groups of evolved populations. Ten genes belonged to this category (Fig. 4B). Four of these genes (DVU1333, DVU1389, DVU2507, and tRNA-leu) were mutated in An-T and EC-T populations. Two genes (DVU2396 and DVU2394) acquired mutations in An-T and ES-T populations. Three genes acquired mutations in at least one population from all three groups. These included a chromate transporter gene (*chrA*; DVU0426), a leucyl-tRNA synthetase gene (*leuS*; DVU1196), and an iron-sulfur cluster-binding protein gene (DVU3028). Only one phage-related tail fiber gene (DVU1486) was mutated in both the EC-T and ES-T populations. The genes that acquired mutations in populations from two or more groups in phase II were dependent on the starting genotype (Fisher exact test, *P < *0.001). This trend held true even considering just the genes that were mutated in phase II in at least one population from both groups with prior experimenter-driven evolution (the EC-T and the ES-T groups; Fisher exact test, *P* = 0.028). These results demonstrated that the phase II genetic changes were influenced by the starting genotype.

### Parallel mutations acquired during phase II evolution.

A total of 17 genes were mutated in two or more populations within the same group (Fig. 4B). The numbers of these parallel mutations in the ES-T, EC-T, and An-T groups were 10, 6, and 1, respectively. Interestingly, all six EC-T populations acquired mutations in heat shock protein *hspC* (DVU2441). EC-T 3 and EC-T 4 acquired unique nonsense mutations that led to truncation. EC-T 6 acquired two mutations with one missense mutation that led to an isoleucine-to-valine substitution and a 4-bp deletion that resulted in a frameshift with an 8-amino-acid addition. Three EC-T populations had mutations in the promoter region, with identical mutations in EC-T 1 and EC-T 2 and a different mutation in EC-T 5. In contrast, each instance of parallel mutations at the gene level in the ES-T group (10 genes) occurred in just two of the populations. The predicted function of these genes included energy production and conversion (DVU2383 and DVU3348), amino acid metabolism and transport (DVU0930 and DVU2084), lipid metabolism (DVU0424 and DVU1207), transcription (DVU1519), signal transduction (DVU1427), and unknown function (DVU1722 and DVU3092). The one gene that acquired a mutation in two or more populations within the An-T group was DVU1208, which corresponds to the fatty acid/phospholipid synthesis protein *plsX* gene.

### Functional analysis of mutations acquired during phase II of evolution.

Genetic convergence was also able to occur at a higher level if two or more genes corresponding to similar functions were mutated in different populations. Therefore, we evaluated the genetic changes using Gene Ontology (GO) enrichment analysis and the DAVID database, with the DvH NCBI NC_002937 reference genome used as the background (42, 43). However, no significantly enriched GO terms were found for any of the three groups. We then categorized all of the mutated genes in the nonmutator populations based on classification using the cluster of orthologous groups (COGs) (44) (data acquired from the MicrobesOnline database) (45) to compare the selections of functional targets among the three groups. Many of the genes thus categorized encode hypothetical proteins and are therefore categorized as “Function Unknown.” Most of the remaining genes for each group fall into the “Signal Transduction Mechanisms” category (“T”). This is unsurprising, as transcriptional regulators and two-component systems have been previously shown to be major targets of selection during evolution experiments (46–49). Beyond the T category, several differences existed among the three groups (Fig. 4C; see also Table S4). Notable differences include a larger number of mutations in genes from both the “Cell wall/membrane/envelope biogenesis” (“M”) category and the “Posttranslational modification, protein turnover, chaperones” (“O”) category for populations in the EC-T group. Additionally, the An-T and ES-T groups were found to have multiple mutations in the “Energy production and conversion” (“C”) category whereas the EC-T group had only one. On the other hand, the An-T and EC-T groups had more mutations in the “Inorganic ion transport and metabolism” (“P”) category than the ES-T group. Overall, the COGs mutated during phase II differed from one group to the other (Fisher exact test, *P* ≤ 0.001). Additionally, Fisher exact tests performed for the three pairwise comparisons between the groups revealed that the COGs mutated in the EC-T group were significantly different from those in the ES-T group (Bonferroni-corrected *P* ≤ 0.001). However, there was no significant difference in the COGs mutated between the An-T group and either the EC-T or ES-T group. It appears that the two groups with prior genetic divergence adopted different functional strategies to accommodate elevated temperature but share common strategies with the An group.

## DISCUSSION

Populations in natural settings often face environments with a variety of stressful conditions that impose multiple selective pressures. Populations diverge genetically and physiologically as they adapt to environmental pressures, but whether these populations employ similar evolutionary solutions in response to a second common stress condition remains unclear. Here, we carried out a two-phase evolution study of three groups of replicate DvH populations to directly assess the influence of prior genetic and phenotypic divergence on evolution in adaptation to a second common environment.

Previous studies have revealed complex and sometimes contradictory pictures of the relationship between prior divergence and phenotypic evolution. A study using the *Tobacco etch virus* found that the specific mutations contributing to infection of a new host were contingent on prior evolutionary conditions but that this did not influence the emergence of an infective phenotype (19). Evolving yeast populations showed evidence of partial genetic convergence leading to convergent phenotypes (18). In contrast, genetic convergence (but with phenotypic divergence) was found in the evolution of E. coli (13). Here, we observed that the initial phenotypic differences (growth rate and fitness) between ES_AN_ and EC_AN_ were mostly eliminated by adaptation to a common novel environment. All but one population showed increased fitness under the evolution condition of 41°C, but the magnitude of fitness change did not seem to be influenced by prior divergence. However, we did observe a slight effect of the previous evolutionary condition on the growth rates of the evolved populations. Both ES_AN_ and EC_AN_ had growth advantages, including higher growth rates and fitness, under conditions of elevated temperature (Fig. 2), which implied less selection pressure in phase II than was the case with the An-T group. Beneficial mutations acquired in a higher-fitness background can have a smaller fitness effect due to interactions among mutations, which is referred to as “diminishing-returns epistasis” (50, 51). Therefore, phase II mutations arising in the EC-T and ES-T populations may have been less beneficial than those in the An-T populations which had a lower-fitness background. Therefore, the evolutionary trajectory could have been influenced by prior evolution even if the final phenotypes were similar. The magnitude of the influence of prior genetic and phenotypic divergence on the adaptation to a common novel environment likely depends on the relationship between the two environments and the numbers of generations in both phases.

Unlike the genetic convergence seen in a two-phase evolution study of E. coli reported previously (13), we observed further genetic divergence in phase II evolution. The overall level of genetic divergence was mostly contributed by 53% of the genes that acquired mutations occurring in only one population. One explanation could be that the results reflected the fact that we had sequenced the entire population at the end of phase II whereas only a single clone from each population was sequenced in the E. coli study (13). Therefore, within-population genetic variations were revealed in this study whereas the previous work likely captured only the most frequent mutations. Nevertheless, a certain level of genetic convergence at both the gene and functional levels was observed but mostly between the An-T group and either the EC-T group or ES-T group. Overall, the mutations that were acquired in the second, novel environment showed signs of historical contingency as there was very little convergence between the EC-T and ES-T groups. One specific example of divergent genetic changes involves the heat shock protein *hspC* (DVU2441). Heat shock proteins are known to be important players in typical bacterial responses to increased temperature, as are other protein turnover and chaperone proteins (33, 52, 53). Surprisingly, mutations in heat shock protein genes were found only in EC-T populations. It is appealing to conjecture that the prior phase of evolution created an opportunity for these mutations to occur in the EC-T group. While the nonsense mutations and frameshift mutations likely resulted in loss of function of the gene, future work is needed to elucidate the roles of these mutations in temperature adaptation. Additionally, examination of a larger number of founding genotypes and replicate populations will help to determine the relationship between the emergence of specific mutations and the prior genetic background.

In addition to the *hspC* gene (DVU2441) involved in protein turnover and chaperone protein activities, genes involved in other functional categories such as energy production and conversion, cell wall synthesis, translation and transcription, lipid metabolism, inorganic ion transport, and signal transduction were also found in mutated form in evolved DvH populations, consistent with previous studies that had investigated the response of both DvH and E. coli to elevated temperature. As expected, the historical environment contributed to the overall differences observed between the EC-T and ES-T groups. Taken together, these results indicate that temperature adaptation is a complex trait and that the prior genetic divergence likely influenced the selection of beneficial mutations.

Overall, our study provided evidence to support the concept that phenotypic convergence can be achieved through diverse genetic changes. The populations in this study that had diverged during phase I evolution displayed similar phenotypes but diverse underlying genetic changes after 1,000 generations at elevated temperature. This could indicate that the populations differ in their locations in the genotype landscape and are climbing to different adaptive peaks. However, future work is needed to determine if these populations will eventually converge or if they will continue to diverge in terms of genetic changes, overall fitness, growth rates, or other traits.

## MATERIALS AND METHODS

### Bacterial strains, growth conditions, and experimental evolution design.

Initially, a clonal isolate (An) of Desulfovibrio vulgaris Hildenborough (DvH, ATCC 29579) was used to found 12 populations (29). Six populations were named EC (evolved control) 1 to EC 6 and cultured in 10 ml of LS4D medium (60 mM lactate and 50 mM sulfate [54]) at 37°C, and the other six populations were named ES (evolved salt) 7 to ES 12 and cultured identically but with the addition of 100 mM NaCl. The populations were transferred every 48 h with a 1-to-100 dilution in anaerobic culture tubes. At 1,200 generations (180 transfers), single-colony isolates were selected for a previous study assessing adaptation to salt stress (29). These two clones were named EC 3–10 (from population EC 3) and ES 9–11 (from population ES 9) and were characterized in detail (29, 30) and were used as a starting point in this study. EC 3–10 (here referred to as EC_AN_ for clarity) was used to start six replicate populations named EC-T (evolved control–temperature) 1 to EC-T 6. ES 9–11 (here referred to as ES_AN_) was used to start six replicate populations named ES-T (evolved salt–temperature) 1 to ES-T 6. In addition, six replicate populations were founded from the original ancestor clone (An) and named An-T (ancestor–temperature) 1 to An-T 6. All 18 populations were propagated for 1,000 generations (150 transfers) in LS4D medium at an elevated temperature of 41°C (phase II). This temperature was chosen as it was shown to induce a moderate stress but still allowed growth. Populations were propagated as described above and archived at −80°C every 100 generations (every 15 transfers). Here, the term “population” is used throughout to describe samples that contain all the variations present at a given generation and the term “clonal isolate” describes a sample that was plated and from which a single colony was selected.

### Growth phenotype tests.

Growth measurements were acquired in triplicate for each population. Cultures were grown in LS4D medium at 41°C, and growth rates were obtained from growth curves generated for each population.

### Whole-genome, whole-population sequencing.

Genomic DNA (gDNA) was extracted from an overnight culture of each population with a GenElute bacterial genomic DNA kit (Sigma-Aldrich, St. Louis, MO) following the manufacturer’s protocol. The quantity of the DNA was determined with a Quant-iT PicoGreen double-stranded DNA (dsDNA) assay kit (Thermo Fisher Scientific, Waltham, MA), and DNA quality was assessed by the use of a NanoDrop spectrophotometer (Thermo Fisher Scientific) followed by gel electrophoresis. A 1-μg volume of DNA from each population was fragmented to ∼300 bp with a Covaris M220 focused ultrasonicator (Covaris, Woburn, MA). Libraries were prepared with a Kapa hyper prep kit (KAPA Biosystems, Wilmington, MA) following the manufacturer’s protocol. Illumina sequencing was done at the Oklahoma Medical Research Foundation (Oklahoma City, OK) with a HiSeq 3000 PE150 system. Populations were sequenced to at least 100-fold coverage of the genomes. Sequence reads were aligned to the DvH reference genome (NC_002937) and to pDV (NC_005863), a native plasmid, and mutation annotations were done with Geneious R9.1 (Biomatters, Newark, NJ) (55). In addition, we analyzed the sequencing data with *breseq* to verify single nucleotide variants (SNVs) and to determine structural variants caused by insertion sequences (IS elements) (56, 57).

### Mutation annotation.

Each mutation was assigned to a gene and mutation type based on the criteria described previously by Good et al. (39). Briefly, each mutation (frequency of at least 10%) was mapped to a DvH reference genome (NC_002937 or NC_005863) and assigned to a gene (including 100 bp upstream of the coding sequence to include putative promoter regions) or categorized as intergenic. The 10% mutation frequency cutoff was chosen to focus on mutations which were under the direct or indirect influence of natural selection, given that at the time of transfer with a population size of *N_e_* of ∼10^7^, new mutations would require ∼10^6^ generations (0.1 *N_e_*) to reach 10% frequency by genetic drift alone (39, 40). SNVs were assigned as nonsense, missense, or synonymous if they resulted in a stop codon, an amino acid change, or no amino acid change, respectively. Insertions or deletions that were <100 bp in length were assigned as indels. Larger rearrangements were annotated as structural variants.

### Selection of fixed SNV as a population marker.

The majority of fitness experiments in microbial evolution studies have utilized a spontaneous mutation that resulted in an auxotrophic phenotype or a fluorescent marker to distinguish between two competing strains (13, 35, 50, 58). However, due to the lack of such a system in DvH, we selected a fixed SNV (100% frequency) from the whole-genome resequencing data for each evolved population that could be used as a molecular marker to distinguish between the ancestor population and the evolved population. The coordinates selected for each An-T population are listed in Table S4 in the supplemental material. Similarly, fixed SNVs were selected from EC_AN_ (coordinate 666481) and ES_AN_ (coordinate 666077) and used as markers to distinguish between the ancestor population and the EC-T populations and ES-T populations, respectively.

### Head-to-head competitions and fitness calculations.

Competitions were carried out in triplicate for each evolved population versus the ancestor (see Fig. S1 in the supplemental material). Cultures were grown to exponential phase and then mixed to reach an approximate 1:1 cell number ratio of the two competitors according to OD_600_ readings. An inoculum of 100 μl was transferred to fresh LS4D medium. The remaining mixture (0 h sample) was immediately spun down and gDNA was extracted as described above. The populations were competed for 48 h at 41°C. The cultures (48 h sample) were then spun down and gDNA was extracted as before. The gDNA was quantified on a NanoDrop spectrophotometer. PCR was carried out with primers designed to amplify ∼150 bp regions centered on the selected fixed SNV marker. The PCR primers contained the sequencing primer, an adapter, and a sample-specific barcode (reverse primer only; Table S5). PCR was performed with AccuPrime *Taq* (Thermo Fisher Scientific) following the manufacturer’s protocol. PCR products were checked on a 1% (wt/vol) agarose gel and quantified by the use of a PicoGreen dsDNA assay kit (Thermo Fisher Scientific). Equal amounts of the samples were pooled and sequenced with an Illumina MiSeq system. Sequencing reads were aligned to the reference sequence by the use of Geneious R9.1, and the ratio of evolved SNV to ancestor SNV was calculated.

10.1128/mBio.00569-20.1 FIG S1 Overview of fitness assay experimental design. The ancestor and an evolved population were grown to the early exponential phase and then mixed 1:1 and used to inoculate fresh LS4D medium. Additionally, gDNA was extracted from the mixture (time 0 h sample). After 48 h at 41°C, gDNA was extracted (time 48 h sample). A fixed mutation was selected from each evolved population for use as a marker to distinguish between DNA derived from the ancestor and DNA derived from the evolved population. PCR was used to amplify the genomic locus containing the fixed single nucleotide variant (SNV) using primers that contained an adapter, a sequencing primer, a sample-specific barcode (reverse primer only), and a gene-specific primer (Table S5). Samples were sequenced with an Illumina MiSeq system to determine the ratio of evolved cells to ancestor cells at both the beginning and the end of the competition. Download FIG S1, TIF file, 1.1 MB. Copyright © 2020 Kempher et al. 2020 Kempher et al. This content is distributed under the terms of the Creative Commons Attribution 4.0 International license.

10.1128/mBio.00569-20.6 TABLE S5 Primer sequences for fitness assay (5′→3′). Download Table S5, DOCX file, 0.01 MB. Copyright © 2020 Kempher et al. 2020 Kempher et al. This content is distributed under the terms of the Creative Commons Attribution 4.0 International license.

Fitness (*W*) was calculated as described previously by Lenski et al. (58). Briefly, the ratio of Malthusian parameters (ratio of the logarithm of the final cell numbers over initial cell numbers) of an evolved strain and the ancestor strain was determined as they competed head to head according to the equation *W* = ln(*E*_48h_/*E*_0h_)/ln(*A*_48h_/*A*_0h_), where *E* represents the number of cells of the evolved population and *A* is the number of cells of the ancestor (58). Total cell numbers for the 0-h samples and the 48-h samples were determined by plate counts. The SNV ratio determined by Illumina sequencing was used to calculate *E*_0h,_ *E*_48h_, *A*_0h_, and *A*_48h_ from the total cell number at times 0 h and 48 h.

### Statistical analyses.

Populations An-T 1, An-T 2, and ES-T 4 appeared to have developed a mutator phenotype, and mutators typically allow the accumulation of neutral mutations that can complicate identifying signatures of adaptation (59). Therefore, we performed statistical analyses for phenotypic assays with and without the mutators. The effects of history (between-group difference) and chance (within-group difference) were calculated using a nested ANOVA with linear mixed-effect models (LMM) (60, 61). In the LMM, the difference of means between groups was treated as the fixed effect and within-group differences were treated as the random effect. Genetic similarity was evaluated with Bray-Curtis dissimilarity at the gene level (using nonsynonymous mutations) as described previously (35). This metric takes into consideration each gene that acquired a mutation and the frequency of each mutation. Values range from 0 to 1, where 0 represents two populations with no commonly mutated genes and 1 represents two populations with identical mutational gene profiles. All statistical analyses were performed in R v 3.4.4 with vegan library v2.5.

### Data availability.

The whole-genome, population sequencing data were deposited in the NCBI SRA database under BioProject identifier (ID) PRJNA610762.
