# Supplementary material for: Effects of Genetic and Physiological Divergence on the Evolution of a Sulfate-Reducing Bacterium under Conditions of Elevated Temperature
Source: mBio. 2020 Aug 18;11(4):e00569-20. doi: 10.1128/mBio.00569-20 (PMC7439460; doi:10.1128/mBio.00569-20)
Supplement: TABLE S1 [file mBio.00569-20-st001.docx]

| **Table S1. Pre-existing mutations in the Ancestor DvH compared to the reference *Desulfovibrio vulgaris* Hildenborough genome from NCBI (NC_002937).^1^** | | | | |
| --- | --- | --- | --- | --- |
| Coordinate | Affected Gene | Nucleotide Change | Amino Acid Change | Type |
| 42868 | intergenic | 1:G | No | Ins |
| 211389 | -39 (putative promoter region) of DVU0170, methyl accepting chemotaxis protein | 1:A | No | Ins |
| 882517 | intergenic | -1:C | No | Del |
| 1144620 | intergenic | 1:C | No | Ins |
| 1191169 | DVU1087, hypothetical protein | -1:C | 73 more amino acids | Del |
| 1313342 | intergenic | 1:C | No | Ins |
| 1773256 | DVU1698, hypothetical protein | G → C | Gln→Glu | SNV |
| 1773346 | DVU1698, hypothetical protein | -1:A | truncated protein | Del |
| 1897096 | DVU1831, transporter | 1:T | 147 more amino acids | Ins |
| 1913197 | DVU1842, putative lipoprotein | T→G | Asp→Ala | SNV |
| 2083308 | DVU2001, site-specific recombinase, phage integrase family | 1:C | 243 more amino acids | Ins |
| 2982788 | intergenic | 2:CC | No | Ins |
| 3056212 | DVU2950, sensory box protein/GGDEF | 1:G | 400 amino acids | Ins |
| 3064064 | DVU2995, hypothetical protein | C→G | A→G | SNV |
| 3064065 | DVU2995, hypothetical protein | G→C | A→G | SNV |
| 3066926 | -33 (putative promoter region) of DVU2690, sigma-54 dependent transcriptional regulator | 3:ACG | No | Ins |
| 3140577 | DVU3022, sensory box histidine kinase/response regulator | -2:CA | 6 amino acid changes | Del |
| 3140596 | DVU3022, sensory box histidine kinase/response regulator | 2:GA | 6 amino acid changes | Ins |
| 3142198 | DVU3023, *atoC,* response regulator | A→G | D→G | SNV |
| 3276864 | DVU3129, hypothetical protein | 1:C | 22 more amino acids | Ins |
| 3455727 | DVU3280, peptide ABC transporter, ATP-binding protein | 1:T | 228 amino acids | Ins |
| 3457155 | intergenic | 3:CGC | No | Ins |
| ^1^The table was constructed based on data from A. Zhou, K. L. Hillesland, Z. He, W. Schackwitz, et al., ISME J 9:2360 –2372, 2015, https://doi.org/10.1038/ismej.2015.45 | | | | |
| Abbreviations- Del: deletion; Ins: insertion; SNV: single nucleotide variant. | |  |  |  |
|  | | | | |
