# Supplementary material for: Effects of Genetic and Physiological Divergence on the Evolution of a Sulfate-Reducing Bacterium under Conditions of Elevated Temperature
Source: mBio. 2020 Aug 18;11(4):e00569-20. doi: 10.1128/mBio.00569-20 (PMC7439460; doi:10.1128/mBio.00569-20)
Supplement: TABLE S2 [file mBio.00569-20-st002.docx]

| **Table S2. Polymorphic loci in ancestral DvH and selected nucleotide in EC_AN_ and ES_AN_.^1^** | | | | | | |
| --- | --- | --- | --- | --- | --- | --- |
| Coordinate | Affected Gene | Polymorphism | EC_AN_^2^ | ES_AN_^2^ | AA change for EC_AN_ | AA change for ES_AN_ |
| 2775672 | DVU2664, *ptsB-2*, phosphate ABC transporter | **C**/G | C | G | None | A → P |
| 2381876 | DVU2287, *CooK*, hydrogenase | **T**/G | G | T | U→G | U→S |
| 2381877 | DVU2287, *CooK*, hydrogenase | **G**/C | G | C |  |  |
| 1426830 | DVU1349, *SelGGPS*, geranylgeranyl diphosphate synthase | **T**/G | T | G | None | V→G |
| 2104739 | DVU2023, hypothetical protein | **G**/C | G | C | None | None |
| 2502193 | intergenic | **G**/C | G | C | None | Intergenic |
| 2905203 | DVU2802, transcriptional regulator, GntR family | **G**/A | A | G | A→T | None |
| 3169435 | DVU3045, *fexB*, sensory box histidine, kinase/response regulator | **G**/C | C | G | G→R | None |
| 1599469 | DVU1530, metallo-beta-lactamase family | **C**/T | T | C | None | None |
| 326403 | DVU0281, exopolysaccharide biosynthesis protein | **G**/A | A | G | None | None |
| 535249 | DVU0467, *trpD*, anthranilate phosphoribosyl transferase | **G**/C | C | G | None | None |
| ^1^The table was constructed based on data from A. Zhou, K. L. Hillesland, Z. He, W. Schackwitz, et al., ISME J 9:2360 –2372, 2015, https://doi.org/10.1038/ismej.2015.45 | | | | | | |
| ^2^Grey shading indicates the selected nucleotide is a mutation (SNV) compared to the NCBI reference strain (NC_002937). | | | | | | |
