# Supplementary material for: Effects of Genetic and Physiological Divergence on the Evolution of a Sulfate-Reducing Bacterium under Conditions of Elevated Temperature
Source: mBio. 2020 Aug 18;11(4):e00569-20. doi: 10.1128/mBio.00569-20 (PMC7439460; doi:10.1128/mBio.00569-20)
Supplement: TABLE S3 [file mBio.00569-20-st003.docx]

| **Table S3. Mutations acquired by ES_AN_ and EC_AN_ during Phase I of evolution.^1^** | | | | | | |
| --- | --- | --- | --- | --- | --- | --- |
| Strain | Mutation type | Mutation position | Affected gene(s) | Mutation position | Nucleotide change | Amino-acid change |
| ES_AN_ | SNV | Gene | DVU0597a, *lytS*, regulatory protein, putative regulator of cell autolysis | 666077 | C→ T | T→I |
|  | SNV | Gene | DVU2571a, *feoB*, ferrous iron transport protein B | 2685757 | G → A | A→V |
|  | SNV | Gene | DVU1204a, *fabF*, 3-oxoacyl-(acyl-carrier-protein) synthase II | 1296562 | C → T | G→S |
|  | SNV | Gene | DVU2287a, *cooK*, hydrogenase, CooK subunit, selenocysteine-containing, putative | 2381877 | G → C | U→S |
|  | SNV | Gene | DVU2472, conserved hypothetical protein | 2581002 | G → T | R→L |
|  | SNV | Gene | DVU2664, *pstB-2*, phosphate ABC transporter, ATP-binding protein | 2775672 | C → G | A→P |
|  | SNV | Gene | DVU1349, *selGGPS*, geranylgeranyl diphosphate synthase | 1426830 | T → G | V→G |
|  | SNV | Gene | DVU2023, hypothetical protein | 2104739 | G → C | None |
|  | SNV | Intergenic |  | 2502193 | G → C | non-coding |
|  | Deletion | Gene | DVU1777–DVU1786 | 1842155 to 1849967 | −7813 nt | −10 genes |
|  | Deletion | Gene | DVU1862, GGDEF domain protein | 1930359 | −1: C | 27 amino acids changed |
| EC_AN_ | SNV | Gene | DVU0597b, *lytS*, regulatory protein, putative regulator of cell autolysis | 666481 | C → T | P→S |
|  | SNV | Gene | DVU2571b, *feoB*, ferrous iron transport protein B | 2685839 | A → G | F→L |
|  | SNV | Gene | DVU1204b, *fabF*, 3-oxoacyl-(acyl-carrier-protein) synthase II | 1296677 | C → T | M→I |
|  | SNV | Gene | DVU2287b, *cooK*, hydrogenase, CooK subunit, selenocysteine-containing, putative | 2381876 | T → G | U→G |
|  | SNV | Gene | DVU0942, *fur*, ferric uptake regulator | 1034709 | G → A | E→K |
|  | SNV | Gene | DVU2395, sensor histidine kinase | 2499343 | G → A | Q→stop |
|  | SNV | Gene | DVU0797, conserved hypothetical protein | 883424 | C → T | E→K |
|  | SNV | Gene | DVU0799, conserved hypothetical protein | 885112 | G → A | S→F |
|  | SNV | Gene | DVU2802, transcriptional regulator, GntR family | 2905203 | G → A | A→T |
|  | SNV | Gene | DVU3045, *fexB*, sensory box histidine kinase/response regulator | 3169435 | G → C | G→R |
|  | SNV | Gene | DVU1530, metallo-beta-lactamase family protein | 1599469 | C → T | None |
|  | SNV | Gene | DVU0281, exopolysaccharide biosynthesis protein, putative | 326403 | G → A | None |
|  | SNV | Gene | DVU0467, *trpD*, anthranilate phosphoribosyltransferase | 535249 | G →C | None |
|  | SNV | Intergenic |  | 2207959 | G → C | non-coding |
|  | Deletion | Gene | DVU2349, carbohydrate phosphorylase family protein | 2442657 | −12: GTCCCGAAGCTC | −4 amino acids |
| ^1^The table was constructed based on data from A. Zhou, K. L. Hillesland, Z. He, W. Schackwitz, et al., ISME J 9:2360 –2372, 2015, <https://doi.org/10.1038/ismej.2015.45>.  ^2^Grey shading indicates the mutation originated from a polymorphic locus in the ancestor. | | | | | | |
