# Supplementary material for: Effects of Genetic and Physiological Divergence on the Evolution of a Sulfate-Reducing Bacterium under Conditions of Elevated Temperature
Source: mBio. 2020 Aug 18;11(4):e00569-20. doi: 10.1128/mBio.00569-20 (PMC7439460; doi:10.1128/mBio.00569-20)
Supplement: TABLE S4 [file mBio.00569-20-st004.docx]

| **Table S4. Total mutations acquired for each population (variant frequency ≥10%) during 1000 generations at 41°C.** | | | | | | | |  |  |
| --- | --- | --- | --- | --- | --- | --- | --- | --- | --- |
| **Population** | **Coordinate of Mutation*** | **Affected Gene** | **Gene Annotation** | **COG** | **Mutation Classification** | **Nucleotide Change** | **Amino Acid Change** | **Protein Effect** | **Variant Freq. (%)** |
| **An-T 1** | 2,381,877 | DVU2287 | hydrogenase, CooK subunit, selenocysteine-containing, putative | C | missense | G→C | U→S | Sub | 100.0% |
|  | 1,066,030* | intergenic | - | - | noncoding | T→A | - | - | 100.0% |
|  | 2,104,739 | DVU2023 | hypothetical protein | S | synonymous | G→C | None | None | 99.7% |
|  | 2,505,317 | DVU2401 | hydrogenase, iron-sulfur cluster-binding subunit, putative | C | missense | C→A | D→Y | Sub | 99.4% |
|  | 2,502,193 | intergenic | - | - | noncoding | G→C | - | - | 99.3% |
|  | 2,775,672 | DVU2664 | phosphate ABC transporter, ATP-binding protein, putative | P | missense | C→G | A→P | Sub | 99.1% |
|  | 1,426,830 | DVU1349 | geranylgeranyl diphosphate synthase | H | missense | T→G | V→G | Sub | 98.9% |
|  | 1,647,754 | DVU1564 | hypothetical protein | S | indel | (GCA)_3→2_ | -A | Del | 98.6% |
|  | 2,991,030 | DVU2894 | sigma-54 dependent transcriptional regulator | T | nonsense | C→T | Q→Stop | Trun | 98.3% |
|  | 1556471-1596075 | ~30 genes | phage related (PR3 from Ref) | Var. | SV | - | - | - | 85.0% |
|  | 801,763 | DVU0721 | sensory box histidine kinase | T | indel | Δ1 bp | -433 AA | FS | 82.6% |
|  | 1,588,391 | DVU1518 | transcriptional regulator cI, truncation | K | missense | G→A | H→Y | Sub | 82.5% |
|  | 1,585,455 | DVU1514 | hypothetical protein | S | missense | C→T | D→N | Sub | 79.8% |
|  | 1,564,548 | DVU1486 | phage related, tail fiber protein, truncation | S | missense | T→C | N→S | Sub | 78.4% |
|  | 1,562,230 | DVU1485 | hypothetical protein | S | missense | G→A | S→F | Sub | 77.7% |
|  | 1,565,976 | DVU1488 | minor tail protein, putative | S | missense | C→T | E→K | Sub | 75.8% |
|  | 1,585,966 | DVU1515 | type II DNA modification methyltransferase, putative | L | missense | G→A | P→L | Sub | 74.4% |
|  | 1,585,960 | DVU1515 | type II DNA modification methyltransferase, putative | L | missense | T→G | H→P | Sub | 73.6% |
|  | 1,575,254 | DVU1500 | HK97 family major capsid protein | R | missense | C→T | G→R | Sub | 71.2% |
|  | 885,052 | DVU0799 | hypothetical protein | S | missense | G→T | T→N | Sub | 58.0% |
|  | 2,498,459 | DVU2395 | sensor histidine kinase | T | indel | +T | T→N | FS | 56.4% |
|  | 1,574,993 | DVU1500 | HK97 family major capsid protein | R | missense | G→A | H→Y | Sub | 55.5% |
|  | 1,577,915 | DVU1502 | HK97 family portal protein | S | missense | C→T | R→H | Sub | 46.6% |
|  | 1,289,564 | DVU1196 (*leuS*) | leucyl-tRNA synthetase | J | missense | T→C | D→G | Sub | 36.4% |
|  | 885,148 | DVU0799 | hypothetical protein | S | missense | A→G | L→P | Sub | 32.3% |
|  | 950,266 | DVU0859 | hypothetical protein | S | indel | (G)6 → (G)5 | G→R | FS | 28.2% |
|  | 2,511,391 | DVU2406 | hypothetical protein | S | missense | C→T | C→Y | Sub | 28.0% |
|  | 2,619,724 | DVU2507 (*mraY*) | phospho-N-acetylmuramoyl-pentapeptide-transferase | M | missense | T→G | N→T | Sub | 26.9% |
|  | 2,862,419 | DVU2754 (*qor*) | quinone oxidoreductase | CR | missense | C→T | G→D | Sub | 26.5% |
|  | 885,140 | DVU0799 | hypothetical protein | S | missense | A→T | S→T | Sub | 17.3% |
|  | 1,575,055 | DVU1500 | HK97 family major capsid protein | R | missense | C→T | G→E | Sub | 16.8% |
|  | 1,562,165 | DVU1485 | hypothetical protein | S | missense | A→T | S→T | Sub | 13.2% |
|  | 1,562,163 | DVU1485 | hypothetical protein | S | synonymous | T→G | None | None | 13.2% |
|  | 1,563,103 | DVU1486 | phage related, tail fiber protein, truncation | S | missense | C→T | A→T | Sub | 12.4% |
|  | 1,585,942 | DVU1515 | type II DNA modification methyltransferase, putative | L | indel | Δ12 bp | -147 AA | FS | 12.3% |
|  | 2,499,408 | DVU2395 | sensor histidine kinase | T | indel | Δ11 bp | - 243 AA | FS | 10.0% |
| **An-T 2** | 2,775,672 | DVU2664 | phosphate ABC transporter, ATP-binding protein, putative | P | missense | C→G | A → P | Sub | 100.0% |
|  | 1,930,141* | DVU1862 | GGDEF domain-containing protein | T | nonsense | C→T | Q→Stop | Trun | 100.0% |
|  | 2,381,877 | DVU2287 | hydrogenase, CooK subunit, selenocysteine-containing, putative | C | missense | G→C | U→S | Sub | 99.4% |
|  | 716,874 | intergenic | - | - | noncoding | T→A | - | - | 99.4% |
|  | 1,796,665 | DVU1722 | hypothetical protein | S | synonymous | C→A | none | none | 99.3% |
|  | 2,104,739 | DVU2023 | hypothetical protein | S | synonymous | G→C | none | None | 99.2% |
|  | 2,502,193 | intergenic | - | - | noncoding | G→C | - | - | 98.7% |
|  | 1,426,830 | DVU1349 | geranylgeranyl diphosphate synthase | H | missense | T→G | V → G | Sub | 98.2% |
|  | 2,511,152 | DVU2405 | alcohol dehydrogenase, iron-containing | C | indel | +T | -324 AA | FS | 98.0% |
|  | 1,588,881 | DVU1519 | transcriptional regulator | K | indel | Δ2 bp | -57 AA | FS | 93.2% |
|  | 1590364 | intergenic | - | - | indel | Δ36 bp | - | - | 92.5% |
|  | 1,585,460 | DVU1514 | hypothetical protein | S | missense | T→C | N → S | Sub | 91.4% |
|  | 1,562,299 | DVU1485 | hypothetical protein | S | missense | A→T | F → Y | Sub | 90.8% |
|  | 1,585,918 | DVU1515 | type II DNA modification methyltransferase, putative | L | missense | C→T | G → D | Sub | 87.7% |
|  | 1,564,549 | DVU1486 | phage related, tail fiber protein, truncation | S | missense | T→C | N → D | Sub | 84.0% |
|  | 1,289,111 | DVU1196 (*IeuS*) | leucyl-tRNA synthetase | J | missense | T→C | N → S | Sub | 77.4% |
|  | 665,999 | DVU0597 (*lytS*) | regulatory protein | T | missense | A→G | N → S | Sub | 76.2% |
|  | 96,789 (Plasmid) | DVUA0072 | glycosyl transferase, group 1 family protein | M | missense | G→T | H→N | Sub | 65.3% |
|  | 2,331,467 | DVU2239 | glycosy hydrolase family protein | G | missense | G→A | R → W | Sub | 65.2% |
|  | 1556471-1596075 | ~30 genes | phage related (PR3 from Ref) | Var. | SV | - | - | - | 35.0% |
|  | 1,409,627 | DVU1333 | hypothetical protein | S | missense | A→T | R → S | Sub | 23.7% |
|  | 2,089,097 | DVU2009 | hypothetical protein | S | missense | T → G | W → G | Sub | 20.6% |
|  | 1,800,457 | DVU1728 | hypothetical protein | S | synonymous | T → G | none | none | 16.4% |
|  | 2,619,404 | DVU2507 (*mraY*) | phospho-N-acetylmuramoyl-pentapeptide-transferase | M | missense | G→A | L → F | Sub | 13.6% |
|  | 1,800,559 | DVU1728 | hypothetical protein | S | synonymous | G → A | none | none | 12.1% |
| **An-T 3** | 2,502,193 | intergenic | - | - | noncoding | G → C | - | - | 100.0% |
|  | 2,104,739 | DVU2023 | hypothetical protein | S | synonymous | G → C | None | None | 99.7% |
|  | 2,381,877 | DVU2287 | hydrogenase, CooK subunit, selenocysteine-containing, putative | C | missense | G → C | U→S | Ext | 99.3% |
|  | 2,775,672 | DVU2664 | phosphate ABC transporter, ATP-binding protein, putative | P | missense | C → G | A → P | Sub | 98.8% |
|  | 1,426,830 | DVU1349 | geranylgeranyl diphosphate synthase | H | missense | T → G | V → G | Sub | 98.6% |
|  | 306,982 | DVU0264 | ferredoxin, 4Fe-4S, putative | C | missense | A → T | H → Q | Sub | 93.7% |
|  | 1,409,647 | tRNA-Leu-3 | tRNA with anticodon TAG for Leu | - | noncoding | C → T | - | - | 93.5% |
|  | 2,498,044 | DVU2394 | sigma-54 dependent transcriptional regulator/response regulator | T | missense | T → C | K → R | Sub | 91.3% |
|  | 2,619,495 | DVU2507 (*mraY*) | phospho-N-acetylmuramoyl-pentapeptide-transferase | M | missense | C → T | M → I | Sub | 72.6% |
|  | 1,929,651 | DVU1862 | GGDEF domain-containing protein | T | indel | -A | -262 AA | FS | 64.3% |
|  | 2,089,103 | DVU2009 | hypothetical protein | S | indel | +GG | -6 AA | FS | 21.0% |
|  | 2,089,097 | DVU2009 | hypothetical protein | S | missense | T → G | W → G | Sub | 20.8% |
| **An-T 4** | 2,775,672 | DVU2664 | phosphate ABC transporter, ATP-binding protein, putative | P | missense | C → G | A → P | Sub | 100.0% |
|  | 1,426,830 | DVU1349 | geranylgeranyl diphosphate synthase | H | missense | T → G | V → G | Sub | 100.0% |
|  | 1,034,641* | DVU0942 | FUR family transcriptional regulator | P | missense | A → C | D → A | Sub | 100.0% |
|  | 2,381,877 | DVU2287 | hydrogenase, CooK subunit, selenocysteine-containing, putative | C | missense | G → C | Stop→S | Ext | 98.8% |
|  | 2,502,193 | intergenic | - | - | noncoding | G → C | - | - | 98.8% |
|  | 2,104,739 | DVU2023 | hypothetical protein | S | synonymous | G → C | None | None | 97.6% |
|  | 97,679 (Plasmid) | DVUA0072 | glycosyl transferase, group 1 family protein | M | missense | C→T | R→H | Sub | 94.5% |
|  | 1,409,655 | tRNA-Leu-3 | tRNA with anticodon TAG for Leu | - | noncoding | T → A | - | - | 45.5% |
|  | 2,443,480 | DVU2349 | carbohydrate phosphorylase family protein | G | missense | T → C | L → P | Sub | 29.7% |
|  | 1,930,141 | DVU1862 | GGDEF domain-containing protein | T | nonsense | C → T | Q→Stop | Trun | 24.0% |
|  | 1,287,395 | DVU1196 (*leuS*) | leucyl-tRNA synthetase | J | missense | A → G | L → P | Sub | 22.4% |
|  | 2,497,875 | DVU2394 | sigma-54 dependent transcriptional regulator/response regulator | T | missense | C → A | K → N | Sub | 20.9% |
|  | 977,925 | DVU0887 | transglycosylase, putative | M | synonymous | G→A | None | None | 19.7% |
|  | 266,493 | DVU0231 | hypothetical protein | S | synonymous | T→C | None | None | 19.6% |
|  | 1,409,627 | DVU1333 | hypothetical protein | S | missense | A→T | R→S | Sub | 15.7% |
|  | 16,521 | DVU0013 | sensory box histidine kinase | T | indel | (GCT)_4→3_ | -L | Del | 14.1% |
|  | 2,586,715 | DVU2477 (*ptsS*) | periplasmic phosphate-binding protein PstS | P | synonymous | C→T | None | None | 13.5% |
|  | 1,932,940 | DVU1867 (*dapF*) | diaminopimelate epimerase | E | missense | G→A | E→K | Sub | 12.4% |
|  | 2,500,748 | DVU2396 | alcohol dehydrogenase, iron-containing | C | missense | A→C | F→V | Sub | 12.2% |
|  | 1,421,548 | DVU1349 | geranylgeranyl diphosphate synthase | H | missense | A→G | N→D | Sub | 10.2% |
|  | 2,498,578 | DVU2395 | sensor histidine kinase | T | indel | Δ1 bp | +13 AA | FS | 10.1% |
| **An-T 5** | 2,334,286 | DVU2241 (*pdxA*) | pyridoxal phosphate biosynthetic protein | H | missense | G → A | T → M | Sub | 100.0% |
|  | 2,381,877 | DVU2287 | hydrogenase, CooK subunit, selenocysteine-containing, putative | C | missense | G → C | U→S | Sub | 100.0% |
|  | 2,502,193 | intergenic | - | - | noncoding | G → C | - | - | 100.0% |
|  | 1,034,799* | DVU0942 | FUR family transcriptional regulator | P | missense | A → T | N → Y | Sub | 100.0% |
|  | 2,775,672 | DVU2664 | phosphate ABC transporter, ATP-binding protein, putative | P | missense | C → G | A → P | Sub | 99.3% |
|  | 716,874 | intergenic | - | - | noncoding | T → A | - | - | 99.2% |
|  | 2,104,739 | DVU2023 | hypothetical protein | S | synonymous | G → C | None | None | 98.6% |
|  | 885,145 | DVU0799 | hypothetical protein | S | missense | T → G | N → T | Sub | 98.5% |
|  | 1,426,830 | DVU1349 | geranylgeranyl diphosphate synthase | H | missense | T → G | V → G | Sub | 98.5% |
|  | 2,498,335 | DVU2394 | sigma-54 dependent transcriptional regulator/response regulator | T | missense | A → T | L → Q | Sub | 95.9% |
|  | 1,930,141 | DVU1862 | GGDEF domain-containing protein | T | nonsense | C → T | - | Trun | 95.2% |
|  | 1,299,241 | DVU1208 (*plsX*) | fatty acid/phospholipid synthesis protein PlsX | I | missense | T → G | Stop→Y | Ext | 95.0% |
|  | 666,798 | DVU0597 (*lytS*) | regulatory protein | T | indel | -GGG | GG → G | Del | 61.5% |
|  | 482,752 | DVU0426 (*chrA*) | chromate transport protein ChrA | P | missense | A → T | M → K | Sub | 50.5% |
|  | 1,289,109 | DVU1196 (*leuS*) | leucyl-tRNA synthetase | J | missense | A → T | W → R | Sub | 35.1% |
| **An-T 6** | 2,381,876 | DVU2287 | hydrogenase, CooK subunit, selenocysteine-containing, putative | C | missense | T → G | U→G | Sub | 100.0% |
|  | 1,599,469 | DVU1530 | metallo-beta-lactamase family protein | J | synonymous | C → T | None | None | 100.0% |
|  | 2,498,111 | DVU2394 | sigma-54 dependent transcriptional regulator/response regulator | T | missense | T → C | T → A | Sub | 100.0% |
|  | 2,905,314* | DVU2802 | GntR family transcriptional regulator | K | missense | G → A | D → N | Sub | 100.0% |
|  | 535,249 | DVU0467 (*trpD*) | anthranilate phosphoribosyltransferase | E | synonymous | G → C | None | None | 99.2% |
|  | 1,299,241 | DVU1208 (*plsX*) | fatty acid/phospholipid synthesis protein PlsX | I | missense | T → G | Stop→Y | Ext | 99.2% |
|  | 326,403 | DVU0281 | exopolysaccharide biosynthesis protein, putative | M | synonymous | G → A | None | None | 98.7% |
|  | 2,685,856 | DVU2571 (*feoB*) | ferrous iron transport protein B | P | missense | C → T | G → D | Sub | 98.5% |
|  | 713,660 | DVU0643 | thiF protein, putative | H | noncoding | A → G | - | - | 89.2% |
|  | 173,399 | DVU0138 | response regulator | T | missense | T → A | V → D | Sub | 88.4% |
|  | 482,972 | DVU0426 (*chrA*) | chromate transport protein ChrA | P | missense | T → C | T → A | Sub | 87.3% |
|  | 1,462,324 | DVU1389 | hypothetical protein | S | indel | (G)8 → (G)9 | -190 AA | FS | 86.6% |
|  | 2082649-2129207 | ~46 genes | ISDVU5 region | Var. | SV | - | - | - | 74.6% |
|  | 2,444,488 | DVU2349 | carbohydrate phosphorylase family protein | G | missense | A → G | D → G | Sub | 13.1% |
|  | 3,150,969 | DVU3028 | iron-sulfur cluster-binding protein | C | missense | T → A | M → K | Sub | 12.9% |
|  | 229,869 | DVU0185 | hypothetical protein | S | missense | A → G | Y → C | Sub | 12.5% |
|  | 3,376,132 | DVU3210 (*thrC*) | threonine synthase | E | synonymous | T → C | None | None | 10.1% |
| **EC-T 1** | 1,897,098 | intergenic | - | - | indel (noncoding) | +T | - | - | 99.5% |
|  | 482,162 | DVU0426 (*chrA*) | chromate transport protein ChrA | P | missense | C → T | A → T | Sub | 96.4% |
|  | 1,761,151 | intergenic | - | - | indel (noncoding) | (A)9 → (A)8 | - | - | 95.4% |
|  | 2,619,709 | DVU2507 (*mraY)* | phospho-N-acetylmuramoyl-pentapeptide-transferase | M | missense | G → A | T → M | Sub | 78.3% |
|  | 1,409,627 | DVU1333 | hypothetical protein | S | missense | A → T | R → S | Sub | 77.7% |
|  | 2,602,908 | DVU2491 | ABC transporter, ATP-binding protein | P | indel | (C)8 → (C)9 | -259 AA | FS | 67.9% |
|  | 872,738 | DVU0786 | penicillin-binding protein | M | missense | A→G | L→P | Sub | 17.3% |
|  | 1,288,923 | DVU1196 (*leuS*) | leucyl-tRNA synthetase | J | missense | T→A | M→L | Sub | 15.0% |
|  | 1,929,908 | DVU1862 | GGDEF domain-containing protein | T | missense | T→C | L→P | Sub | 14.2% |
|  | 2,547,447 | DVU2441 | HSP20 family protein | O | noncoding | T→C | - | - | 12.2% |
| **EC-T 2** | 482,162 | DVU0426 (*chrA*) | chromate transport protein ChrA | P | missense | C → T | A → T | Sub | 98.8% |
|  | 1,761,151 | DVU1683 | hypothetical protein | S | indel (noncoding) | (A)9 → (A)8 | - | - | 80.9% |
|  | 2,602,908 | DVU2491 | ABC transporter, ATP-binding protein | P | indel | (C)8 → (C)9 | -259 AA | FS | 80.2% |
|  | 1,409,627 | DVU1333 | hypothetical protein | S | missense | A → T | R → S | Sub | 79.8% |
|  | 2,619,709 | DVU2507 (*mraY*) | phospho-N-acetylmuramoyl-pentapeptide-transferase | M | missense | G → A | T → M | Sub | 73.2% |
|  | 1,288,923 | DVU1196 (*leuS*) | leucyl-tRNA synthetase | J | missense | T→A | M→L | Sub | 17.6% |
|  | 1,347,643 | DVU1260 | outer membrane protein P1, putative | I | indel | Δ1 bp | -99 AA | FS | 12.7% |
|  | 1,347,642 | DVU1260 | outer membrane protein P1, putative | I | indel | Δ1 bp | +3 AA | FS | 12.6% |
|  | 2,547,447 | DVU2441 | HSP20 family protein | O | noncoding | T→C | - | - | 12.5% |
|  | 1,929,908 | DVU1862 | GGDEF domain-containing protein | T | missense | T→C | L→P | Sub | 10.1% |
| **EC-T 3** | 489,742 | intergenic | - | - | indel | (A)8 → (A)9 | - | - | 97.4% |
|  | 1,409,675 | tRNA-Leu-3 | tRNA with anticodon TAG for Leu | - | noncoding | G → A | - | - | 97.3% |
|  | 2,619,343 | DVU2507 (*mraY*) | phospho-N-acetylmuramoyl-pentapeptide-transferase | M | missense | C → T | G → D | Sub | 97.1% |
|  | 2,547,597 | DVU2441 | HSP20 family protein | O | Nonsense | C → T | R→Stop | Trun | 75.1% |
|  | 1,137,203 | DVU1034 | hypothetical protein | S | missense | A → T | M → L | Sub | 62.0% |
|  | 1,347,181 | DVU1260 | outer membrane protein P1, putative | I | indel | Δ1 bp | -250 AA | FS | 19.3% |
|  | 482,845 | DVU0426 (*chrA*) | chromate transport protein ChrA | P | missense | A→G | V→A | Sub | 17.6% |
|  | 1,930,037 | DVU1862 | GGDEF domain-containing protein | T | missense | T→C | L→P | Sub | 13.7% |
|  | 1,413,334 | DVU1337 (*lon)* | ATP-dependent protease La | O | missense | A → G | E → G | Sub | 13.0% |
|  | 3,150,966 | DVU3028 | iron-sulfur cluster-binding protein | C | missense | A→G | E→G | Sub | 12.6% |
|  | 1,818,390 | DVU1750 | hypothetical protein | S | missense | G→A | D→N | Sub | 11.6% |
| **EC-T 4** | 1,409,675 | tRNA-Leu-3 | tRNA with anticodon TAG for Leu | - | noncoding | G → A | - | - | 93.6% |
|  | 1,930,141 | DVU1862 | GGDEF domain-containing protein | T | nonsense | C → T | Q→Stop | Trun | 54.5% |
|  | 2,374,606 | DVU2284 | hypothetical protein | S | missense | T → C | Y → H | Sub | 53.1% |
|  | 2,547,697 | DVU2441 | HSP20 family protein | O | nonsense | T → A | L→Stop | Trun | 50.9% |
|  | 872,211 | DVU0785 (*rodA*) | rod shape-determining protein RodA | D | missense | A → G | V → A | Sub | 42.6% |
|  | 801,075 | DVU0721 | sensory box histidine kinase | T | indel | (G)8 → (G)7 | -771 AA | FS | 40.6% |
|  | 2,230,789 | DVU2134 | hypothetical protein | S | missense | T → C | L → S | Sub | 27.9% |
|  | 2,374,564 | DVU2284 | hypothetical protein | S | missense | G→T | A→S | Sub | 15.9% |
|  | 874,656 | DVU0787 | hypothetical protein | S | missense | A→G | L→P | Sub | 12.8% |
| **EC-T 5** | 1,347,187 | DVU1260 | outer membrane protein P1, putative | I | indel | -CA | -224 AA | FS | 99.3% |
|  | 1,289,475 | DVU1196 (*leuS*) | leucyl-tRNA synthetase | J | missense | A→G | Y→H | Sub | 97.9% |
|  | 1,031,639 | DVU0941 | M16 family peptidase | R | missense | G→A | D→N | Sub | 93.3% |
|  | 2,548,222 | DVU2442 | HSP20 family protein | O | missense | T→C | V→A | Sub | 61.9% |
|  | 872,781 | DVU0786 | penicillin-binding protein | M | missense | T→C | M→V | Sub | 38.1% |
|  | 647,796 | DVU0582 | sensory box histidine kinase | T | missense | C→T | R→H | Sub | 23.9% |
|  | 1,462,324 | DVU1389 | hypothetical protein | S | indel | (G)8 → (G)9 | -190 AA | FS | 21.0% |
|  | 2,613,023 | DVU2501 (*ftsQ*) | cell division protein FtsQ, putative | M | missense | A→G | S→P | Sub | 19.0% |
|  | 1,386,266 | DVU4003 | hypothetical protein | S | missense | A→G | I→T | Sub | 19.0% |
|  | 874,044 | DVU0786 | penicillin-binding protein | M | missense | C→T | A→T | Sub | 16.8% |
|  | 2,547,446 | DVU2441 | HSP20 family protein | O | noncoding | T→C | - | - | 16.1% |
|  | 664,775 | DVU0596 (*lytR*) | DNA-binding response regulator LytR | KT | missense | A→G | K→E | Sub | 13.3% |
|  | 876,524 | DVU0789 (*mreB1)* | rod shape-determining protein MreB | D | missense | G→T | L→I | Sub | 10.0% |
| **EC-T 6** | 801,076 | DVU0721 | sensory box histidine kinase | T | indel | (G)8 → (G)9 | -755 AA | FS | 79.9% |
|  | 1,462,324 | DVU1389 | hypothetical protein | S | indel | (G)8 → (G)9 | -190 AA | FS | 75.7% |
|  | 2,652,469 | DVU2540 | 2-hydroxyglutaryl-CoA dehydratase, D-component | E | missense | T → C | T→A | Sub | 72.8% |
|  | 2,547,510 | DVU2441 | HSP20 family protein | O | missense | A → G | I→V | Sub | 71.0% |
|  | 1,394,565 | DVU1304 (*rplD*) | 50S ribosomal protein L4 | J | synonymous | C → T | None | None | 70.4% |
|  | 1,409,675 | tRNA-Leu-3 | tRNA with anticodon TAG for Leu | - | noncoding | G→A | - | - | 70.3% |
|  | 481,976 | DVU0426 (*chrA*) | chromate transport protein ChrA | P | missense | A → G | S→P | Sub | 69.4% |
|  | 1,562,958 | DVU1486 | phage related, tail fiber protein, truncation | S | missense | T → A | D→V | Sub | 67.9% |
|  | 1,975,162 | DVU1900 | hypothetical protein | S | missense | G → A | R→C | Sub | 53.0% |
|  | 873,423 | DVU0786 | penicillin-binding protein | M | missense | A → G | F→L | Sub | 25.3% |
|  | 2,547,884 | DVU2441 | HSP20 family protein | O | indel | -GAAA | +8 AA | FS | 23.8% |
|  | 2,612,303 | DVU2500 (*ftsA*) | cell division protein FtsA | D | missense | A → G | S→P | Sub | 22.9% |
|  | 1,287,449 | DVU1196 (*leuS*) | leucyl-tRNA synthetase | J | missense | A → G | L→P | Sub | 22.8% |
|  | 2,374,666 | DVU2284 | hypothetical protein | S | missense | A→T | R→W | Sub | 15.4% |
| **ES-T 1** | 2,172,464 | DVU2084 (*appA)* | oligopeptide-binding protein, putative | E | indel (noncoding) | +A | - | - | 100.0% |
|  | 885,140 | DVU0799 | hypothetical protein | S | missense | A→G | S→P | Sub | 100.0% |
|  | 3,151,100 | DVU3028 | iron-sulfur cluster-binding protein | C | missense | T→C | Y→H | Sub | 100.0% |
|  | 1,020,772 | DVU0930 (*proB*) | gamma-glutamyl kinase | E | synonymous | T→C | None | None | 95.9% |
|  | 1,298,382 | DVU1207 (*fabH*) | 3-oxoacyl-(acyl-carrier-protein) synthase III | I | missense | C→T | G→D | Sub | 95.8% |
|  | 665,699 | DVU0597 (lytS) | regulatory protein | T | missense | T→C | L→P | Sub | 95.6% |
|  | 3,235,873 | DVU3092 | hypothetical protein | S | missense | A→G | E→G | Sub | 94.6% |
|  | 1,796,658 | DVU1722 | hypothetical protein | S | missense | T→C | T→A | Sub | 94.4% |
|  | 2,498,056 | DVU2394 | sigma-54 dependent transcriptional regulator/response regulator | T | missense | T→G | D→A | Sub | 94.0% |
|  | 1,562,919 | DVU1486 | phage related, tail fiber protein, truncation | S | missense | G→A | A→V | Sub | 58.4% |
|  | 1,563,028 | DVU1486 | phage related, tail fiber protein, truncation | S | missense | G→A | R→C | Sub | 56.5% |
|  | 1,588,428 | DVU1599 (*crcB*) | CrcB protein, camphor resistance | D | noncoding | A→G | - | - | 56.5% |
|  | 1556471-1596075 | ~30 genes | phage related (PR3 from Ref) | Var. | SV | - | - |  | 45.0% |
|  | 883,604 | DVU0797 | hypothetical protein | S | nonsense | G→A | Q→Stop | Trun | 27.7% |
|  | 1,590,361 | intergenic | - | - | indel | Δ38 bp | - | - | 26.0% |
| **ES-T 2** | 3,151,100 | DVU3028 | iron-sulfur cluster-binding protein | C | missense | T→C | Y→H | Sub | 98.0% |
|  | 2,498,056 | DVU2394 | sigma-54 dependent transcriptional regulator/response regulator | T | missense | T→G | D→A | Sub | 97.6% |
|  | 2,172,464 | DVU2084 | oligopeptide-binding protein, putative | E | indel (noncoding) | +A | - | - | 97.5% |
|  | 1,298,382 | DVU1207 (*fabH*) | 3-oxoacyl-(acyl-carrier-protein) synthase III | I | missense | C→T | G→D | Sub | 97.3% |
|  | 885,140 | DVU0799 | hypothetical protein | S | missense | A→G | S→P | Sub | 97.2% |
|  | 1,796,658 | DVU1722 | hypothetical protein | S | missense | T→C | T→A | Sub | 96.1% |
|  | 665,699 | DVU0597 (*lytS*) | regulatory protein | T | missense | T→C | L→P | Sub | 95.6% |
|  | 1,020,772 | DVU0930 (*proB*) | gamma-glutamyl kinase | E | synonymous | T→C | None | None | 95.1% |
|  | 3,235,873 | DVU3092 | hypothetical protein | S | missense | A→G | E→G | Sub | 94.4% |
|  | 1,563,028 | DVU1486 | phage related, tail fiber protein, truncation | S | missense | G→A | R→C | Sub | 68.4% |
|  | 1,562,919 | DVU1486 | phage related, tail fiber protein, truncation | S | missense | G→A | A→V | Sub | 64.0% |
|  | 1,588,428 | DVU1519 | transcriptional regulator | K | noncoding | A→G | - | - | 63.3% |
|  | 1556471-1596075 | ~30 genes | phage related (PR3 from Ref) | Var. | SV | - | - |  | 58.0% |
|  | 883,604 | DVU0797 | hypothetical protein | S | nonsense | G→A | Q→Stop | Trun | 41.4% |
|  | 1590361-1590339 | intergenic | - | - | indel | Δ38 bp | - | - | 26.0% |
| **ES-T 3** | 458,685 | intergenic | - | - | noncoding | A→C | - | - | 99.5% |
|  | 1,501,689 | DVU1427 | response regulator | T | missense | T→G | K→T | Sub | 98.4% |
|  | 2,203,518 | DVU2109 (*mrp*) | MTH1175-like domain family protein | D | missense | T→C | V→A | Sub | 79.5% |
|  | 885,508 | DVU0799 | hypothetical protein | S | missense | T→A | D→V | Sub | 75.5% |
|  | 2,500,235 | DVU2396 | alcohol dehydrogenase, iron-containing | C | nonsense | G→A | Q→Stop | Trun | 75.5% |
|  | 883,604 | DVU0797 | hypothetical protein | S | nonsense | G→A | Q→Stop | Trun | 75.1% |
|  | 3,150,969 | DVU3028 | iron-sulfur cluster-binding protein | C | missense | T→A | M→K | Sub | 73.1% |
|  | 1590244-1590369 | DVU1521 | hypothetical protein | S | SV | - | - | - | 57.9% |
|  | 1,588,428 | DVU1519 | transcriptional regulator | S | noncoding | A→G | - | - | 57.3% |
|  | 1,564,299 | DVU1486 | phage related, tail fiber protein, truncation | S | missense | G→A | P→L | Sub | 54.6% |
|  | 1556471-1596075 | ~30 genes | phage related (PR3 from Ref) | Var. | SV | - | - |  | 54.0% |
|  | 1,562,230 | DVU1485 | hypothetical protein | S | missense | G→A | S→F | Sub | 52.9% |
|  | 1,562,919 | DVU1486 | phage related, tail fiber protein, truncation | S | missense | G→A | A→V | Sub | 51.8% |
|  | 1,716,784 | DVU1633 | PTS system, IIB component | G | missense | T→G | W→G | Sub | 45.8% |
|  | 885,110 | DVU0799 | hypothetical protein | S | missense | A→T | W→R | Sub | 27.4% |
|  | 480,023 | DVU0424 (*cls*) | cardiolipin synthetase | I | missense | C→T | P→S | Sub | 26.5% |
|  | 1,559,977 | DVU1480 | hypothetical protein | S | missense | C→G | T→R | Sub | 23.0% |
|  | 2,498,532 | DVU2395 | sensor histidine kinase | T | indel | Δ1 bp | +13 AA | FS | 22.2% |
|  | 2,484,786 | DVU2383 | TonB dependent receptor domain-containing protein | C | missense | T →G | L→R | Sub | 20.8% |
|  | 903,882 | DVU0815 (*asmA*) | AsmA family protein | M | synonymous | A→G | - | None | 14.9% |
| **ES-T 4** | 665,371 | DVU0597 (*lytS*) | regulatory protein | T | indel (noncoding) | (C)7→(C)8 | - | - | 95.7% |
|  | 1,738,259 | DVU1656 (*folK*) | 2-amino-4-hydroxy-6-hydroxymethyldihydropteridine pyrophosphokinase | H | missense | T→C | Stop→Q | Ext | 95.5% |
|  | 1,786,693 | DVU1711 | hypothetical protein | S | indel (noncoding | (C)9→(C)8 | - | - | 94.5% |
|  | 3,151,101 | DVU3028 | iron-sulfur cluster-binding protein | C | missense | A→G | Y→C | Sub | 94.0% |
|  | 2,497,021 | DVU2394 | sigma-54 dependent transcriptional regulator/response regulator | T | indel | -AGTGC | +34 AA | FS | 93.6% |
|  | 1,501,346 | DVU1427 | response regulator | T | missense | T→C | I→M | Sub | 93.1% |
|  | 551,025 | DVU0483 (*mutL*) | DNA mismatch repair protein | L | indel | +TGCA | -280 AA | FS | 92.4% |
|  | 1,119,049 | DVU1017 | ABC transporter, ATP-binding protein/permease protein | V | indel | (G)7→(G)8 | -86 AA | FS | 90.9% |
|  | 2,476,624 | DVU2379 | M16 family peptidase putative | R | indel | (C)7→(C)8 | -894 AA | FS | 88.9% |
|  | 2,342,097 | DVU2246 | S1 RNA-binding domain-containing protein | K | missense | C→T | P→S | Sub | 77.0% |
|  | 2,486,318 | DVU2384 | ABC transporter, periplasmic substrate-binding protein | E | indel | (C)9→(C)10 | -132 AA | FS | 76.0% |
|  | 662,190 | intergenic | - | - | indel | (G)9→(G)8 | - | - | 73.1% |
|  | 1,741,278 | DVU1661 | hypothetical protein | S | indel (noncoding) | (G)9→(G)10 | - | - | 71.6% |
|  | 2,438,909 | DVU2343 | amino acid ABC transporter, ATP-binding protein | E | nonsense | G→A | Q→Stop | Trun | 70.6% |
|  | 1,077,922 | DVU0981 | multiphosphoryl transfer protein, putative | G | missense | G→A | G→E | Sub | 68.2% |
|  | 272,190 | DVU0238 | hypothetical protein | S | missense | C→T | R→K | Sub | 63.9% |
|  | 385,020 | DVU0339 (*serA*) | D-isomer specific 2-hydroxyacid dehydrogenase family protein | CH | missense | C→T | M→I | Sub | 62.9% |
|  | 1,299,209 | DVU1207 (*fabH*) | 3-oxoacyl-(acyl-carrier-protein) synthase III | I | noncoding | A→C | - | - | 58.6% |
|  | 2,131,199 | DVU2055 (*metG*) | methionyl-tRNA synthetase | J | missense | C→T | G→R | Sub | 19.2% |
|  | 1,298,518 | DVU1207 (*fabH*) | 3-oxoacyl-(acyl-carrier-protein) synthase III | I | missense | G→A | R→C | Sub | 16.6% |
|  | 2,438,846 | DVU2343 | amino acid ABC transporter, ATP-binding protein | E | missense | T→C | T→A | Sub | 13.5% |
|  | 903,887 | DVU0815 (*asmA*) | AsmA family protein | M | missense | C→G | V→L | Sub | 25.8% |
| **ES-T 5** | 2,497,744 | DVU2394 | sigma-54 dependent transcriptional regulator/response regulator | T | nonsense | A→T | L→Stop | Trun | 99.5% |
|  | 3,194,533 | DVU3062 | sensor histidine kinase/response regulator | T | missense | C→T | G→R | Sub | 97.8% |
|  | 775,106 | intergenic | - | - | indel | (G)7→(G)8 | - | - | 96.8% |
|  | 1,078,607 | DVU0981 | multiphosphoryl transfer protein, putative | G | missense | G→T | E→D | Sub | 93.2% |
|  | 482,162 | DVU0426 (*chrA*) | chromate transport protein ChrA | P | missense | C→T | A→T | Sub | 92.1% |
|  | 806,505 | intergenic | - | - | noncoding | G→A | - | - | 79.2% |
|  | 1,654,664 | DVU1571 (*rho*) | transcription termination factor Rho | K | missense | G→A | A→V | Sub | 76.9% |
|  | 3,520,295 | DVU3348 | pyruvate ferredoxin/flavodoxin oxidoreductase, beta subunit | C | missense | T→C | Q→R | Sub | 71.4% |
|  | 2,686,075 | DVU2571 (*feoB*) | ferrous iron transport protein B | P | missense | A→G | I→T | Sub | 58.9% |
|  | 3,151,101 | DVU3028 | iron-sulfur cluster-binding protein | C | missense | A→G | Y→C | Sub | 15.9% |
|  | 2,497,015 | DVU2394 | sigma-54 dependent transcriptional regulator/response regulator | T | missense | A→G | I→V | Sub | 13.1% |
|  | 571,224 | DVU0500 (*selB*) | selenocysteine-specific translation elongation factor | J | noncoding | T→G | - | - | 10.8% |
| **ES-T 6** | 3,538,337 | DVU3367 (*aspS*) | aspartyl-tRNA synthetase | J | missense | A→G | F→S | Sub | 99.4% |
|  | 1,501,689 | DVU1427 | response regulator | T | missense | T→G | K→T | Sub | 72.2% |
|  | 489,742 | intergenic | - | - | indel | (A)8→(A)9 | - | - | 46.4% |
|  | 883,646 | DVU0797 | hypothetical protein | S | missense | A→C | W→G | Sub | 42.9% |
|  | 2,484,374 | DVU2383 | TonB dependent receptor domain-containing protein | C | nonsense | A→T | K→T | Trun | 26.0% |
|  | 2,497,419 | DVU2394 | sigma-54 dependent transcriptional regulator/response regulator | T | indel | (G)7→(G)8 | -146 AA | FS | 24.3% |
|  | 3,524,417 | DVU3353 (*coaBC*) | phosphopantothenoylcysteine decarboxylase | H | synonymous | C→T | None | None | 20.8% |
|  | 479,282 | DVU0424 (*cls*) | cardiolipin synthetase | I | missense | G→A | A→T | Sub | 19.9% |
|  | 480,023 | DVU0424 (*cls*) | cardiolipin synthetase | I | missense | C→A | P→T | Sub | 19.9% |
|  | 3,519,630 | DVU3347 | pyruvate ferredoxin/flavodoxin oxidoreductase family protein | C | missense | C→T | G→D | Sub | 18.1% |
|  | 1,287,227 | DVU1196 (*leuS*) | leucyl-tRNA synthetase | J | missense | A→G | L→P | Sub | 16.4% |
|  | 3,519,950 | DVU3348 | pyruvate ferredoxin/flavodoxin oxidoreductase, beta subunit | C | missense | A→G | V→A | Sub | 16.1% |
|  | 2,497,039 | DVU2394 | sigma-54 dependent transcriptional regulator/response regulator | T | missense | G→A | S→L | Sub | 15.5% |
|  | 3,397,881 | DVU3230 | flagellar synthesis regulator FleN | D | missense | A→T | V→E | Sub | 13.3% |
|  | 755,683 | DVU0681 | sensor histidine kinase/response regulator | T | missense | A→G | V→A | Sub | 12.6% |
|  | 3,151,097 | DVU3028 | iron-sulfur cluster-binding protein | C | missense | T→C | S→P | Sub | 10.1% |
|  |  |  |  |  |  |  |  |  |  |
| *Single nucleotide variant (SNV) used for fitness assays | | | |  |  |  |  |  |  |
| Gray shading indicates the mutation originated from a polymorphic loci in the ancestor | | | |  |  |  |  |  |  |
| Abbreviations: Ext: Extension; FS: Frameshift; SV: Structural Variant; Sub: Substitution; Trun: Truncation; Var: various; - indicates Not Applicable. | | | | | |  |  |  |  |
