# Supplementary figures and images for: Effects of Genetic and Physiological Divergence on the Evolution of a Sulfate-Reducing Bacterium under Conditions of Elevated Temperature
Source: mBio. 2020 Aug 18;11(4):e00569-20. doi: 10.1128/mBio.00569-20 (PMC7439460; doi:10.1128/mBio.00569-20)

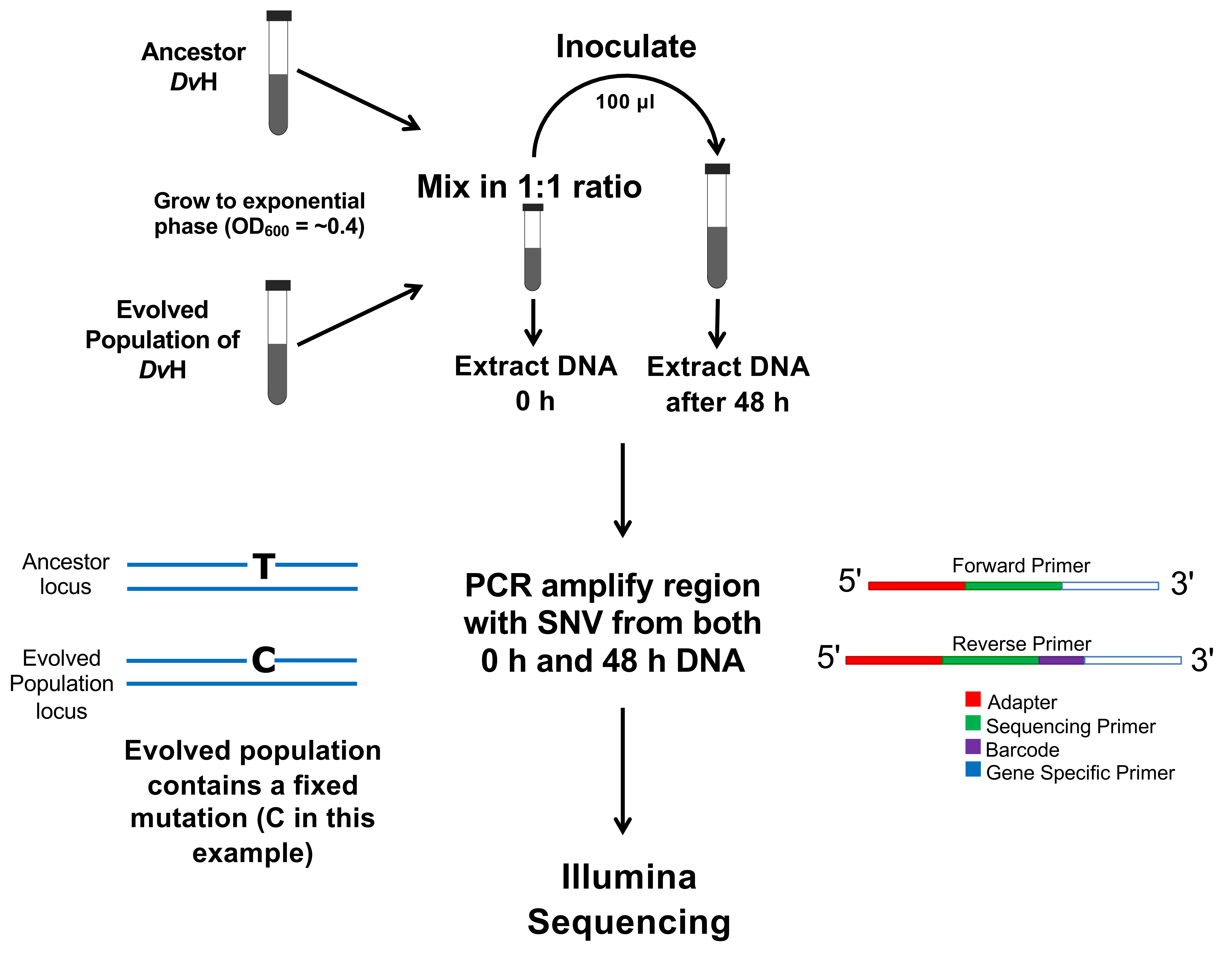

Supplement: FIG S1 [file mBio.00569-20-sf001.tif]
