# Supplementary material for: Effects of Genetic and Physiological Divergence on the Evolution of a Sulfate-Reducing Bacterium under Conditions of Elevated Temperature
Source: mBio. 2020 Aug 18;11(4):e00569-20. doi: 10.1128/mBio.00569-20 (PMC7439460; doi:10.1128/mBio.00569-20)
Supplement: TABLE S5 [file mBio.00569-20-st005.docx]

| **Table S5. Primer Sequences for Fitness Assay (5'→3')** | |
| --- | --- |
| **Forward Adapter** | AATGATACGGCGACCACCGAGATCT |
| **Forward Sequencing Primer** | ACACTCTTTCCCTACACGACGCTCTTCCGATCT |
| **Reverse Adapter** | CAAGCAGAAGACGGCATACGAGAT |
| **Reverse Sequencing Primer** | GTGACTGGAGTTCAGACGTGTGCTCTTCCGATCT |
|  |  |
| **Population** | **Gene Specific Primer** |
| **An-T 1** | Forward: ATCTGACCGCGCCAGAAG |
|  | Reverse: CCGAGAAATGCCGCACCT |
| **An-T 2** | Forward: GGATGGGCGTAGGTTCCG |
|  | Reverse: GGCGTCTCGTCATGGTCTG |
| **An-T 4** | Forward: AAGGTGACGCCGCAGCGGAT |
|  | Reverse: CTTCTGCTCGTAACGCGCCACACC |
| **An-T 5** | Forward: AGGTCTCGCCAAGGAAGTGCATTTCGG |
|  | Reverse: GCGCAGATGCCATAGAGATACATCCGGTG |
| **An-T 6** | Forward: TGGGTCCCGCACTGGTGGAA |
|  | Reverse: CCTCCTCGGCACTGTGCTCCA |
| **EC-T Group** | Forward: GCCACGCAGATCGAACTCGAAGAC |
|  | Reverse: TCGAGAATGAGCTCGCGAGCC |
| **ES-T Group** | Forward: AACCGCACGCTCCCGCAC |
|  | Reverse: TGTGGTGGTCGGAGGCCG |
